# Telomere-to-telomere genome assembly of bitter melon (*Momordica charantia* L. var. *abbreviata* Ser.) reveals fruit development, composition and ripening genetic characteristics

**DOI:** 10.1093/hr/uhac228

**Published:** 2022-10-11

**Authors:** Anzhen Fu, Yanyan Zheng, Jing Guo, Donald Grierson, Xiaoyan Zhao, Changlong Wen, Ye Liu, Jian Li, Xuewen Zhang, Ying Yu, Hong Ma, Qing Wang, Jinhua Zuo

**Affiliations:** Institute of Agri-food Processing and Nutrition, Beijing Academy of Agricultural and Forestry Sciences, Beijing Key Laboratory of Fruits and Vegetable Storage and Processing, Key Laboratory of Vegetable Postharvest Processing of Ministry of Agriculture and Rural Areas, Beijing 100097, China; Beijing Engineering and Technology Research Center of Food Additives, Beijing Advanced Innovation Center for Food Nutrition and Human Health, School of Food and Health, Beijing Technology and Business University (BTBU), Beijing, 100048, China; Institute of Agri-food Processing and Nutrition, Beijing Academy of Agricultural and Forestry Sciences, Beijing Key Laboratory of Fruits and Vegetable Storage and Processing, Key Laboratory of Vegetable Postharvest Processing of Ministry of Agriculture and Rural Areas, Beijing 100097, China; Ministry of Education Key Laboratory of Biodiversity Sciences and Ecological Engineering and State Key Laboratory of Genetic Engineering, Institute of Biodiversity Sciences and Institute of Plant Biology, School of Life Sciences, Fudan University, 2005 Songhu Road, Shanghai 200438, China; School of Biosciences, University of Nottingham, Sutton Bonington Campus, Loughborough, Leicestershire, LE12 5RD, United Kingdom; Institute of Agri-food Processing and Nutrition, Beijing Academy of Agricultural and Forestry Sciences, Beijing Key Laboratory of Fruits and Vegetable Storage and Processing, Key Laboratory of Vegetable Postharvest Processing of Ministry of Agriculture and Rural Areas, Beijing 100097, China; Institute of Agri-food Processing and Nutrition, Beijing Academy of Agricultural and Forestry Sciences, Beijing Key Laboratory of Fruits and Vegetable Storage and Processing, Key Laboratory of Vegetable Postharvest Processing of Ministry of Agriculture and Rural Areas, Beijing 100097, China; Beijing Engineering and Technology Research Center of Food Additives, Beijing Advanced Innovation Center for Food Nutrition and Human Health, School of Food and Health, Beijing Technology and Business University (BTBU), Beijing, 100048, China; Beijing Engineering and Technology Research Center of Food Additives, Beijing Advanced Innovation Center for Food Nutrition and Human Health, School of Food and Health, Beijing Technology and Business University (BTBU), Beijing, 100048, China; Biomarker Technologies Corporation, Beijing 101300, China; Biomarker Technologies Corporation, Beijing 101300, China; Penn State Univ, Huck Inst Life Sci, Dept Biol, University Pk, PA 16802 USA; Institute of Agri-food Processing and Nutrition, Beijing Academy of Agricultural and Forestry Sciences, Beijing Key Laboratory of Fruits and Vegetable Storage and Processing, Key Laboratory of Vegetable Postharvest Processing of Ministry of Agriculture and Rural Areas, Beijing 100097, China; Institute of Agri-food Processing and Nutrition, Beijing Academy of Agricultural and Forestry Sciences, Beijing Key Laboratory of Fruits and Vegetable Storage and Processing, Key Laboratory of Vegetable Postharvest Processing of Ministry of Agriculture and Rural Areas, Beijing 100097, China

## Abstract

*Momordica charantia* L. var. *abbreviata* Ser. (Mca), known as bitter gourd or bitter melon, is a *Momordica* variety with medicinal value and belongs to the Cucurbitaceae family. In view of the lack of genomic information on bitter gourd and other *Momordica* species and to promote Mca genomic research, we assembled a 295.6-Mb telomere-to-telomere (T2T) high-quality Mca genome with six gap-free chromosomes after Hi-C correction. This genome is anchored to 11 chromosomes, which is consistent with the karyotype information, and comprises 98 contigs (N50 of 25.4 Mb) and 95 scaffolds (N50 of 25.4 Mb). The Mca genome harbors 19 895 protein-coding genes, of which 45.59% constitute predicted repeat sequences. Synteny analysis revealed variations involved in fruit quality during the divergence of bitter gourd. In addition, assay for transposase-accessible chromatin by high-throughput sequencing and metabolic analysis showed that momordicosides and other substances are characteristic of Mca fruit pulp. A combined transcriptomic and metabolomic analysis revealed the mechanisms of pigment accumulation and cucurbitacin biosynthesis in Mca fruit peels, providing fundamental molecular information for further research on Mca fruit ripening. This report provides a new genetic resource for *Momordica* genomic studies and contributes additional insights into Cucurbitaceae phylogeny.

## Introduction

**Figure 1:**
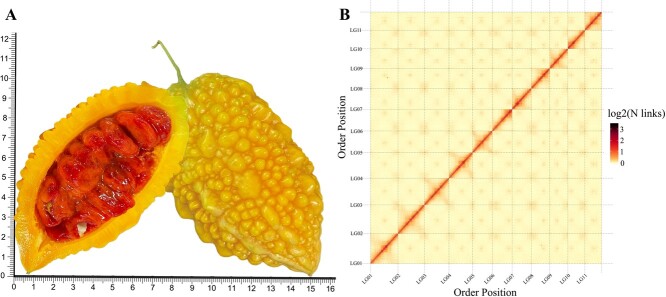
Overview of the Mca genome. (A) Longitudinal section of ripening stage Mca fruit; the unit of the *x* and *y* axes is centimeter. (B) Hi-C heat map of chromosome interactions. The *x* and *y* axes represent the ordered positions of the chromosomes of the genome.

*Momordica charantia* L. var. *abbreviata* Ser. (Mca) is a variety of bitter gourd in the Cucurbitaceae family. Bitter gourd is native to Africa but has been present in Asia for a long period [1, 2]. This Mca variety is mainly distributed in Southern Asia, Southeast Asia and China [3]. It is referred to as ‘Jin ling zi’ or ‘Lai pu tao’ in Chinese and was predominantly grown during the Ming and Qing dynasties [4]. Mca fruits are smaller than those of *M. charantia* L. (MC). During the process of fruit ripening, the fruit peel turns from green to yellow/orange at maturity, the pulp turns from bitter white to sweet red (Fig. 1A), and the fruit is both edible and applicable to ornamental use [5]. Terpenoids play essential roles in the bitterness of *Momordica* fruit. New norcucurbitane triterpenoids have been isolated from the fruit of *Momordica* in recent years [6–8]. The antioxidant and antibacterial effects of these fruits have also attracted attention [9, 10] and suggest that these fruits have potential applications in the health food industry and for controlling food-borne pathogens.

The fruits of many members of the Cucurbitaceae are edible, and the family includes economically important crop species such as cucumber, watermelon, and bitter gourd [11]. Genome assembly is a great tool for plant breeding and crop improvement. With the development of deep sequencing, the genomes of 12 species of Cucurbitaceae have been assembled, namely those of cucumber [12], watermelon [13, 14], melon [15–17], pumpkin [18], sponge gourd [19, 20], bitter gourd [3, 21], wax gourd [22], snake gourd [23], bottle gourd [24, 25], and chayote [26]. All of the species with assembled genomes produce cultivated fruit and, and, with respect to members of the genus *Momordica*, there are wild relatives that potentially contain valuable germplasm. The first version of the bitter gourd OHB3-1 genome was drafted in 2016 [21], but the genes were not anchored to chromosomes. Cui *et al*. [2] reported the first chromosome-level bitter gourd genome, and Matsumura *et al*. [3] published a long-read genome and provided new insights into bitter gourd genetic diversity and domestication. However, to date no Mca genomic information is available.

To promote the progress of Cucurbitaceae and Momordica genomic research, we assembled an Mca genome through third-generation circular consensus sequencing (CCS) as well as high-throughput chromosome conformation capture (Hi-C) sequencing and chromosome assignments. The resulting draft genome was analyzed to identify repeat sequences and coding genes. The evolutionary relationship and gene variations between Mca and other Cucurbitaceae species were indicated by comparative genomic and variation analysis. On the basis of an assay for transposase-accessible chromatin (ATAC) with high-throughput sequencing, transcriptome sequencing and metabolome analysis, Mca genes associated with fruit texture, pigmentation, bitterness, phytohormone synthesis, and signal transduction were identified. Metabolites such as saponins and momordicins and other characteristic substances were analyzed. This assembly of the Mca genome helps improve the understanding of Cucurbitaceae and Mca phylogeny and provides insights into the fruit ripening process.

## Results

### Genome assembly

A 350-bp library was constructed using Mca genomic DNA and was sequenced and filtered on the Illumina sequencing platform, generating 21.28 Gb of high-quality data. The total sequencing depth was ~74.31×. The *k*-mer depth corresponding to the first peak was 30.7 (Supplementary Data Fig. S1), and the calculated length of a single genome was ~286.40 Mb (Table 1). According to the *k*-mer distribution, the estimated repeat content constituted ~25.60%, and the estimated heterozygosity was ~0.12%. These data indicate that Mca has a simple genome, which is conducive to the construction of subsequent fine genomic maps.

**Table 1:** Statistics of the assembly of the Mca genome.

**Parameter**	**Value**
Estimated genome	286.40 Mb
CCS assembly genome (contig length)	299 033 464 bp
Contig number	98
Hi-C assembly	296 021 231 bp
Contig N50	25 379 967 bp
Scaffold N50	25 379 967 bp
Chromosome-anchored length	295 646 031 bp

A PacBio library was constructed using Mca genomic DNA, and ~31 088 764 945 bp of clean data were obtained after sequencing. The total sequencing depth was ~103.96× and the read N50 was 11.9 kb. After assembly, 98 contigs with an N50 value of 25.38 Mb and a GC content of 35.92% were obtained (Table 1). The Core Eukaryotic Genes Mapping Approach (CEGMA) and Benchmarking Universal Single-Copy Orthologs (BUSCO) database evaluation results corresponded to 98.69% and 98.02%, respectively. A total of 1582 complete BUSCOs were detected, with only 22 missing (Supplementary Data Table S1).

**Figure 2:**
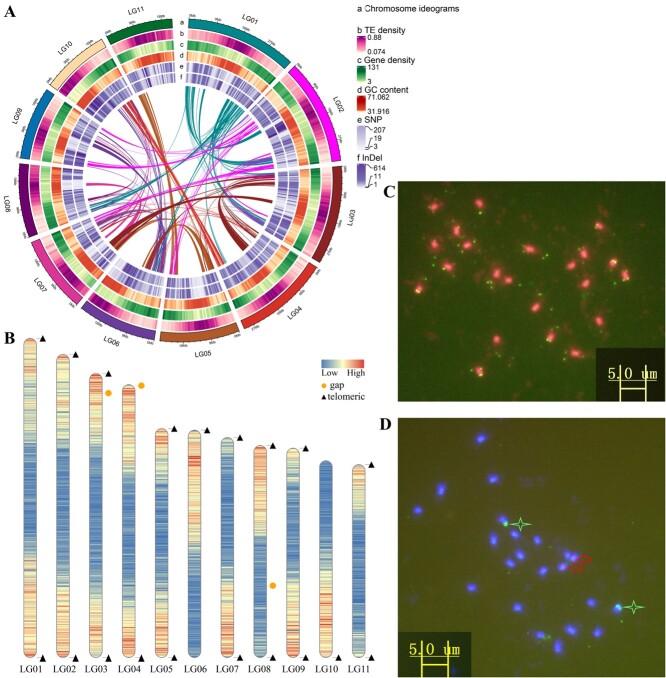
High-quality telomere-to-telomere (T2T) genome assembly and karyotype verification. (A) Genome information, comprising chromosome ideogram information (a), TE density (b), gene density (c), GC content (d), SNPs (e), and InDel (f) variants. Every term in b, c, and d represents 100 kb, and the lines in circles represent chromosomal collinearity. (B) Telomere detection map. The triangles and orange circles represent telomeres and gaps within the assembled chromosomes, red colors indicate high gene density, and blue colors indicate low gene density. (C) Results of telomere and rDNA *in situ* hybridization of telomere repeats (green). (D) Fluorescence *in situ* hybridization (green stars represent hybridization signal indicating 18S rDNA, and red arrows represent hybridization signal indicating 5S rDNA).

A heat map of Hi-C-assembled chromosomes indicated that the genome assembly was complete (Fig. 1B). A total length of 295.6 Mb anchored to 11 chromosomes; this accounted for 99.87% of the assembled genome and can determine direction. The final genome assembly statistics were as follows: 25.4 Mb for contig N50 and scaffold N50, with 98 contigs and 95 scaffolds (Table 1). The gene density, transposable element (TE) content, and GC content are shown in Fig. 2A. Using Tandem Repeats Finder (4.07b) [27], we identified centromeric sequences from the assembled genomes, and the most abundant tandem repeat is the centromere DNA [28]. Using the seven-base telomeric repeat (CCCTAAA at the 5′ end or TTTAGGG at the 3′ end) as a sequence query, we identified 18 telomeres. The candidate centromere tandem repeats, predicted centromeric regions, and telomere regions are shown in Supplementary Data Tables S2, S3, and S4, respectively. With respect to the anchored chromosomes, we found that LG01, LG02, LG05, LG06, LG07, LG09, LG10, and LG11 were assembled as gapless chromosomes, and six chromosomes had integrated telomeres, namely, LG01, LG02, LG05, LG07, LG09, and LG11 (Fig. 2B). Only three chromosome assemblies had gaps, indicating that the assembled genome can be considered a high-quality telomere-to-telomere (T2T) genome. Chromosomes with telomeres and centromere regions are shown in Supplementary Data Fig. S2. Karyotype analysis indicated that Mca has 22 chromosomes with length 1.5–2.0 μm, and they are nearly central or proximal centromeric chromosomes. The telomeres and rDNA were subjected to fluorescence *in situ* hybridization, which showed green signals at the telomeres of each chromosome (Fig. 2C). In Fig. 2D two chromosomes show strong 5SrDNA hybridization signals (red arrows) and another two chromosomes show strong 18SrDNA hybridization signals (green stars). To evaluate genome assembly quality, Merqury [29] results showed that the integrity of the genome assembly was 99.6%, QV = 45.2, and the error rate was only 0.003%, indicating that a genome with high integrity and accuracy was constructed. Detailed assessment results and *k*-mer multiplicity are shown in Table 2 and Supplementary Data Fig. S3, respectively. This high-quality gapless genome can be used to elucidate the mystery of these ‘dark matter’ regions.

**Table 2:** Evaluation of Mca genome assembly quality.

**Assembly**	**All-reads set**	**Solid *k*-mers in the assembly**	**Total solid *k*-mers in the read set**	**Completeness (%)**
*Momordica charantia* L. var. *abbreviate* Ser.	All	218 702 647	219 568 057	99.6059
**Assembly**	***k*-mers uniquely found only in the assembly**	***k*-mers found in both assembly and the read set**	**QV**	**Error rate**
*M. charantia* L. var. *abbreviate* Ser.	171 361	299 031 700	45.2044	0.00301689%

### Genome annotation analysis

Repeated sequences, pseudogenes, non-coding RNAs, and homologous coding genes and functions were annotated using the non-redundant (NR) protein sequence, evolutionary genealogy of genes: Non-supervised Orthologous Groups (eggNOG), Gene Ontology (GO), Kyoto Encyclopedia of Genes and Genomes (KEGG), SwissProt, Pfam, and TrEMBL (Table 3) databases. In total, 45.59% of the sequences composing the Mca genome were predicted to be repeated sequences, consisting mainly of tandem repeats, interspersed repeats and TEs. Based on the constructed repeat database, an ~127-Mb TE sequence was obtained, accounting for 42.47% of the repeated DNA (Supplementary Data Table S5). In addition, 1536 kb of full-length long terminal repeat (LTR) sequences were obtained, including gypsy (15.07%), copia (7.72%), caulimovirus (0.14%), and other sequences (Supplementary Data Table S5). The predicted tandem repeat sequences accounted for 9.34 Mb, or 3.12% (Supplementary Data Table S6). A total of 20 068 genes and 83 pseudogenes were predicted based on homology, transcriptome, and *ab initio* predictions (Supplementary Data Table S7). A total of 99.14% of the obtained genes could be annotated from the databases (Table 3). Most gene annotations being based on transcriptome and genome homology predictions indicated a high degree of confidence. In total, 1581 tRNAs, 1233 rRNAs, and 100 miRNAs were identified. After motif annotation, 948 motifs and 22 204 domains were found in the Mca genome.

### Comparative genomic analysis

**Figure 3:**
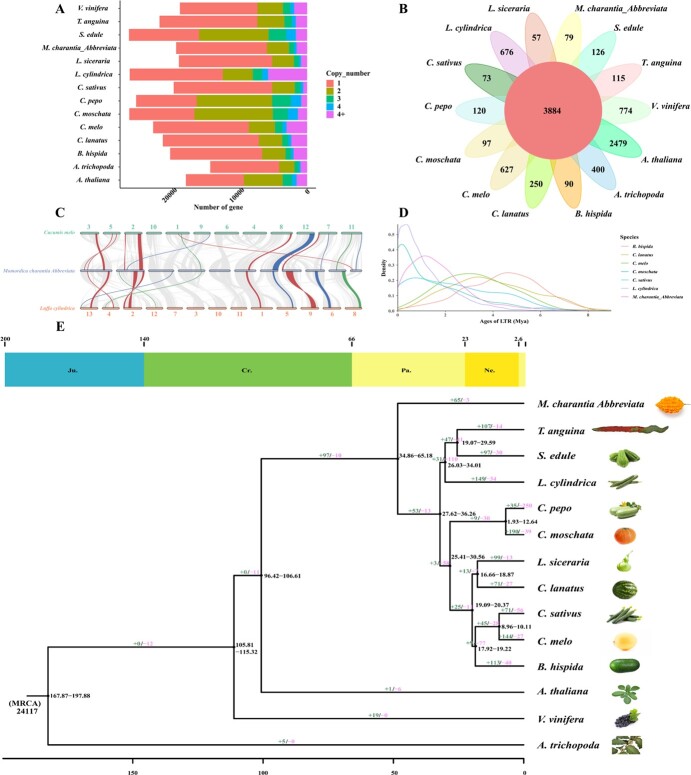
Comparative genomic analysis and genome evolution. (A) Copy number distribution of all gene families in 15 species (*C. moschata*, *Lagenaria siceraria*, *A. trichopoda*, *T. anguina*, *Cucurbita pepo*, *A. thaliana*, *Vitis vinifera*, *B. hispida*, *C. lanatus*, *S. edule*, *C. sativus*, *Cucumis melo*, *L. cylindrica*, Mca). (B) Gene family cluster petal map. The middle circle represents common gene families and the outer petals show specific gene families. (C) Collinearity diagram including *C. melo*, *L. cylindrica*, and Mca*.* Lines in red indicate phytohormones, lines in blue indicate the carotenoid pathway, and lines in green indicate cucurbitacin synthesis. (D) Analysis of LTR insertion times in *B. hispida*, *C. lanatus*, *C. melo*, *C. moschata*, *C. sativus*, *L. cylindrica*, *M. charantia*, and Mca. (E) Evolutionary tree showing the relationships among 15 species with differentiation time. Included are the numbers of contracted gene families (pink color) and expanded gene families (green color). The top of the tree is the absolute age (millions of years) and geological time (Jurassic, Cretaceous, Paleogene, and Neogene).

Thirty-one Mca gene families and 3680 families common to all species were investigated. A total of 1518 specific gene families were detected in bitter gourd; the others are described in Fig. 3B. GO and KEGG pathway enrichment analysis was performed for the Mca-specific gene family members. KEGG pathway analysis indicated that Mca-specific gene family members were associated with RNA polymerase, sesquiterpenoid, and triterpenoid biosynthesis, purine metabolism, and photosynthesis-antenna proteins (Supplementary Data Fig. S4). A total of 1019 single-copy genes were detected, and the gene copy number distribution for all gene families for 15 analyzed species is shown in Fig. 3A. A rooted tree was constructed based on the 1019 single-copy genes, with the outgroup represented by *Amborella trichopoda* (Fig. 3E). This showed that Mca diverged from bitter gourd 0.53–52.49 million years ago (Mya) and that bitter gourd evolved gradually from other Cucurbitaceae crop species ~115.08–120.42 Mya. The contraction and expansion of Mca gene families relative to those of their ancestors were subsequently predicted (Fig. 3E). Sixty-two expanded genes and 106 contracted genes compared with those in bitter gourd were predicted. KEGG pathway enrichment revealed that the expanded genes were associated with photosynthesis, phenylalanine metabolism, and diterpenoid biosynthesis (Supplementary Data Fig. S5A) and that the contracted genes were associated with linoleic acid metabolism and diterpenoid biosynthesis (Supplementary Data Fig. S5B).

Collinearity analysis showed that the percentage of collinear genes between Mca and melon was 55.87%, followed by 54.07% between Mca and sponge gourd (Fig. 3C). Several collinear genes that played roles in fruit ripening and flavor formation during the domestication process, including genes related to phytohormones, the carotenoid pathway and cucurbitacin synthesis, were detected in these three species (Fig. 3C; Supplementary Data Table S8). To further determine the evolutionary relationships of Mca and other Cucurbitaceae gourds, we used the synonymous mutation rate (*K*_s_) and 4-fold synonymous third-codon transversion (4DTv) curves to identify the occurrence of whole-genome duplication (WGD) events. The *K*_s_ peaks for Mca, bitter gourd, and sponge gourd were ~1.32 (Supplementary Data Fig. S6A), and have been verified as corresponding to an ancient genome-wide triploidization (WGT; gamma) event [30]; additionally, these findings indicate that no recent WGD events have occurred. This conclusion is consistent with the 4DTv curves (Supplementary Data Fig. S6B). On the other hand, as shown in Fig. 3D, the insertion time of LTR transposons in Mca, bitter gourd, wax gourd, watermelon, melo, pumpkin, cucumber, and sponge gourd occurred at ~1.14, 1.14, 4.59, 3.35, 3.05, 0.15, 0.70, and 0.35 Mya, respectively.

**Table 3:** Gene functional annotation statistics.

Annotation database	Number of annotations	Annotation percentage
GO_Annotation	17 102	85.22
KEGG_Annotation	15 361	76.54
KOG_Annotation	11 393	56.77
Pfam_Annotation	17 701	88.21
Swissprot_Annotation	16 571	82.57
TrEMBL_Annotation	19 881	99.07
eggNOG_Annotation	17 465	87.03
nr_Annotation	19 864	98.98
All_Annotated	19 895	99.14

### Genome-wide identification of variation

The Mca genome was used as a reference to assess the genome variations that occurred in bitter gourd throughout evolution. This revealed 211 329 single-nucleotide polymorphism (SNP) differences and 100 187 insertions–deletions (InDels), namely 43 085 insertions and 57 102 deletions. Figure 2A shows the SNP and InDel variation site density of Mca and bitter gourd. A total of 210 copy number variations (CNVs) were found in the bitter gourd genome compared with the Mca genome, with 99 copies that increased in number in bitter gourd and 111 that were lost. Other types of genomic structural variation (SV), including long segment chromosome inversions (INVs), chromosome translocations (TRANSs), and duplications (DUPs), were found to have occurred during the process of Mca evolution from bitter gourd; specifically, 374 SVs, namely 39 TRANSs, 18 INVs, and 317 DUPs, were detected. GO enrichment analysis indicated associations with proteolysis among biological processes and polygalacturonase (PG) activity among molecular functions. KEGG pathway analysis indicated associations with sesquiterpenoid and triterpenoid biosynthesis, monoterpenoid biosynthesis, and isoquinoline alkaloid biosynthesis. The SVs affected genes encoding terpene synthase 10 (*TPS10*), polygalacturonase (*PG*), flavonol synthase (*FLS*) and others (Table 4, Supplementary Data Table S6), which associated with phytohormone, texture and terpene metabolize.

**Table 4:** Partial annotation of gene SVs and their absence in PAVs.

Gene ID	SVs	Gene ID	PAVs, absences
Mch09G012590.1	*Flavonol synthase*	Mch04G015270.1	*Squalene monooxygenase*
Mch02G010850.1	*Histidine kinase 3*	Mch10G002190.1	*WRKY12*
Mch01G018830.1	*l-Ascorbate oxidase*	Mch01G011270.1	*Beta-amyrin synthase*
Mch05G008250.1	*Linoleate 9S-lipoxygenase 4*	Mch09G003650.1	*Caffeic acid 3-O-methyltransferase*
Mch03G009990.1	*Gibberellin 2-beta-dioxygenase 8*	Mch01G016490.1	*Gibberellin 3-beta-dioxygenase 3*
Mch10G000480.1	*Terpene synthase 10*	Mch10G000480.1	*Terpene synthase 10*
Mch05G016980.1	*1-Aminocyclopropane-1-carboxylate oxidase*	Mch03G013270.1	*1-Aminocyclopropane-1-carboxylate oxidase-1*

Note: SVs, structural variations; PAVs, presence–absence variations.

In this analysis, the presence of a gene was defined as a sequence of >50 bp with no variation from the reference genome sequence. The absence of a sequence of >50 bp identical to one in the reference genome was defined as deletion variation (absence). We found 572 and 706 sequences that were present and absent, respectively, from the bitter gourd genome. KEGG pathway annotation of the genes present in bitter gourd indicated an association with thiamine metabolism, inositol phosphate metabolism, and nitrogen metabolism. Absent genes were related to sesquiterpenoid and triterpenoid biosynthesis, glycerolipid metabolism, and glycerophospholipid metabolism, which may play roles in Mca evolution. Genes such as *phospholipase D* (*PLD*), *squalene monooxygenase* (*ERG1*), *1-aminocyclopropane-1-carboxylate oxidase-1* (*ACO1*) and *gibberellin 3-beta-dioxygenase 3* (*GA3OX3*) were predicted to be in the group whose members were absent from bitter gourd, which was unique in Mca; this lack of genes may contribute to the phenotypic characteristics of Mca.

### ATAC-sequencing analysis

**Figure 4:**
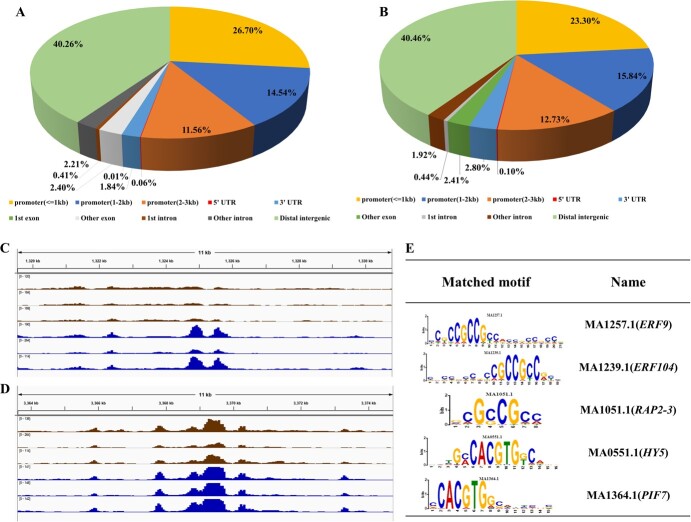
Differentially accessible region (DAR) distance annotation and integrated genome viewer (IGV) visual analysis of DARs and motifs. Distance between DARs and gene elements in IM versus BR fruit (A) and the BR-stage fruit versus RS fruit (B), including distal intergenic areas within 1 kb from the promoter, 1–2 kb from the promoter, 2–3 kb from the promoter, and others. (C) IGV analysis of DARs (*NCED2*) and matched TFs (E) in the IM fruit (brown color) versus BR-stage fruit (blue color) comparison group. The length of this chromosome fragment is 11 kb. (D) IGV analysis of DARs (*WRI1*) and matched TFs (E) in BR-stage fruit (brown color) versus RS fruit (blue color) comparison group. The length of this chromosome fragment is 11 kb; shown are E motif logos of several TFs, generated through MEME and Tomtom.

ATAC-sequencing (ATAC-seq) can be carried out to determine open chromatin regions. Open regions of chromatin contain transcription factor-binding sites (TFBSs), which play important roles in the regulation of gene expression mediated by transcription factors (TFs). Therefore, identifying the representative motif (overrepresented) sequences enriched in the peak region helps us speculate about the potential TFs binding to the sequences corresponding to the peak region. According to our results, pulp tissue was used for ATAC-seq. Sequences with different peaks are also known as differentially accessible regions (DARs). Comparison of samples of immature green (IM) fruit versus breaker (BR)-stage fruit, which show the first signs of color change, revealed 7713 DARs, namely, 6248 increased DARs (corresponding to peaks with increased accessibility in BR-stage fruit compared with IM fruit) and 1465 decreased DARs (corresponding to peaks with decreased accessibility in BR-stage fruit compared with IM fruit). In the BR-stage fruit versus ripening stage (RS) fruit comparison group, 3858 DARs were found, namely, 3222 increased DARs and 636 decreased DARs. Relationships (in terms of distance) between DARs and functional elements of genes in the genome were predicted, such as promoters, 5′ untranslated regions (UTRs), 3′ UTRs, distal intergenic regions, and others, as shown in Fig. 4A and B, the results of which showed that most DARs were distributed in distal intergenic regions and that the second regions were 1 kb from the promoters of genes in both groups. The known TF information obtained from motif annotations can be combined with genes associated with certain peaks, providing a new direction for the study of transcriptional regulation. TFs that bind to open chromatin regions, such as *RAP2-3* (MA1051.1), *ERF9* (MA1257.1), *ERF104* (MA1239.1), *ARF16* (MA1688.1), *PIF7* (MA1364.1), *PIF1* (MA0552.1), *HY5* (MA0551.1), and *BPC6* (MA1402.1), were analyzed in the two comparison groups, as described in Fig. 4C–E and Supplementary Data Fig. S7, and the associated positional information is presented in Supplementary Data Table S9. In the IM fruit versus BR-stage fruit comparison group, the partial position of chromosome 6 included DARs associated with genes involved in the carotenoid pathway and phytohormone signaling, such as *9-cis-epoxycarotenoid dioxygenase NCED2* (*NCED2*), *auxin response factor 18* (*ARF18*), *ethylene-responsive transcription factor 4* (*ERF4*), and *ethylene-responsive transcription factor 060* (*ERF060*; Fig. 4C, Supplementary Data Fig. S5). The chromatin open region including *9-cis-epoxycarotenoid dioxygenase NCED2* (*NCED2*) was matched with TFs such as *BPC6* (MA1402.1), *PIF7* (MA1364.1), *PIF1* (MA0552.1), *HY5* (MA0551.1), and *ERF9* (MA1257.1; Fig. 4C, Supplementary Data Table S10). In the BR-stage fruit versus RS fruit comparison group, essential coding genes, such as *ethylene-responsive transcription factor WRI1* (*WRI1*), *ethylene-responsive transcription fa*ctor *ERF118* (*ERF118*), *auxin response factor 1* (*ARF1*; Fig. 4D, Supplementary Data Fig. S5, Supplementary Data Table S10), were anchored to chromosome 3. The chromosome position coding *WRI1* contained *RAP2-3* (MA1051.1), *ERF9* (MA1257.1), *ERF104* (MA1239.1), *ARF16* (MA1688.1), *PIF7* (MA1364.1), *PIF1* (MA0552.1), *HY5* (MA0551.1), and *BPC6* (MA1402.1; Fig. 4D and E, Supplementary Data Table S10).

### Metabolomic analysis

In fruit pulp tissue, 261 differentially expressed metabolites (DEMs) were detected in pre-ripe fruit (IM fruit versus BR-stage fruit). Specifically, 51 DEMs were increased and 210 DEMs were decreased**.** These compounds were classified as carboxylic acids and derivatives, fatty acids, organooxygen compounds, and others (Supplementary Data Table S11). Bergaptol and 2-propylglutaric acid presented the maximum (69 530-fold) and minimum (1.91e^−6^-fold) fold-changes (FCs), respectively. The metabolites whose abundance increased included amino acids; peptides; analogs such as *N*-methylglycine (6641-fold), l-aspartic acid (1.73-fold), l-ornithine (1.73-fold), and betaine (1.73-fold); and other compounds, such as citric acid* (1.69-fold, * represents an isomer) and quinic acid (3.77-fold; Supplementary Data Table S11). Lipids and lipid-like molecules, such as 2-dodecenedioic acid (0.37-fold), γ-linolenic acid* (0.59-fold), and α-linolenic acid* (0.56-fold; Supplementary Data Table S11) decreased in abundance. In addition, the contents of l-fucose, malonic acid, jasmonic acid, benzoic acid and retinol (vitamin A1) were similarly regulated (Supplementary Data Table S11).

**Figure 5:**
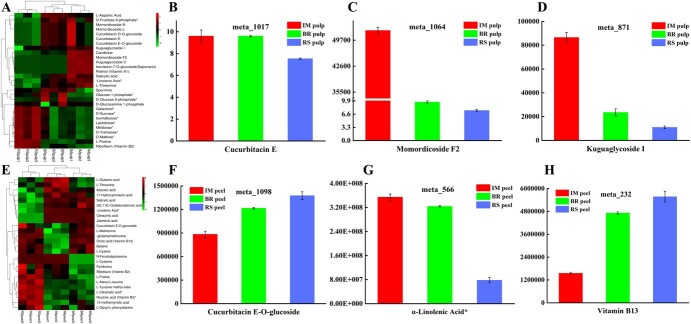
Cluster heat maps and essential metabolites in Mca fruit pulp and peel tissues of the BR versus RS comparison group. (A) Cluster heat map of essential DEMs in the BR-stage pulp versus RS pulp comparison group. (E) Cluster heat map of essential DEMs in the BR-stage peel versus RS peel comparison group. (B–D, F–H) Metabolite counts of cucurbitacin E (B), momordicoside F2 (C), and kuguaglycoside I (D) in the BR-stage pulp versus RS pulp comparison group and cucurbitacin E-O-glucoside (F), α-linolenic acid^*^ (G), and vitamin B13 (H) in the BR-stage peel versus RS peel comparison group.

A total of 376 DEMs were found in the postripening stage (BR-stage fruit versus RS fruit): 129 DEMs were increased, and 247 DEMs were decreased. These DEMs were classified as carboxylic acids and derivatives, fatty acids, organooxygen compounds and others (Supplementary Data Fig. S8A, Supplementary Data Table S12). During the RS, the abundance of substances unique to Cucurbitaceae, such as cucurbitacin E (Fig. 5B), cucurbitacin D-O-glucoside, cucurbitacin E-O-glucoside, momordicoside F2 (Fig. 5C), kuguaglycoside I (Fig. 5D), momordicine IV and momordicoside L, changed by 0.78-fold (Fig. 5A, Supplementary Data Table S12). Other metabolites whose abundance decreased included l-aspartic acid, γ-linolenic acid*, l-threonine, saponarin, salicylic acid, spermine, and jasmonic acid, and metabolites whose abundance increased included l-proline riboflavin (vitamin B2) and oxalic acid (Fig. 5A, Supplementary Data Table S12). In addition, KEGG annotation indicated an association with α-linolenic acid metabolism and monobactam biosynthesis (Supplementary Data Fig. S8B).

In the fruit peel tissue, 200 DEMs were detected in the preripening stage (IM peel versus BR-stage peel), of which 150 DEMs were increased and 50 DEMs were decreased. These DEMs were classified as carboxylic acids and derivatives, fatty acids, benzene derivatives, organooxygen compounds and others (Supplementary Data Table S13). Among them, the content of kaempferol-6,8-di-C-glucoside increased by 59 369-fold and arachidic acid presented the greatest FC decrease. The abundance of amino acids, peptides, and analogs changed significantly; for example, the contents of l-methionine (1.7-fold), l-cystine (2.7-fold), and betaine (2.34-fold) increased, and those of l-glutamic acid (0.35-fold), l-proline (0.44-fold), and l-threonine (0.33-fold) decreased. In addition, major substances involved in this process were detected. Specifically, cucurbitacin E-O-glucoside (Fig. 5F), orotic acid (vitamin B13; Fig. 5H), oxalic acid, l-malic acid, and ricinoleic acid increased in abundance, and pyridoxine (vitamin B6), riboflavin (vitamin B2), abscisic acid, and arachidic acid decreased in abundance (Supplementary Data Table S13).

During the postripening stage (BR-stage fruit versus RS fruit), 192 DEMs were found, namely 88 increased DEMs and 104 decreased DEMs. They were classified as carboxylic acids and derivatives, fatty acids, benzene and substituted derivatives, and organooxygen compounds (Supplementary Data Fig. S8C, Supplementary Data Table S14). Among these DEMs, *N*-feruloylputrescine was the most increased metabolite, and citraconic acid was the least increased (Supplementary Data Table S14). Moreover, with respect to lipids and lipid-like molecules, the contents of citraconic acid (5.34e^−06^-fold), linoleic acid (0.68-fold), (9*Z*,11*E*)-octadecadienoic acid (0.64-fold), α-linolenic acid* (0.24-fold; Fig. 5G), 12-oxo-10*E*-dodecenoic acid (7.14e^−05^-fold), and γ-linolenic acid (0.26-fold) decreased, whereas those of pentadecanoic acid (1.22-fold), 13-methylmyristic acid (1.29-fold), 2,4-dihydroxybenzoic acid* (3.17-fold), and vitamin B13 increased (Fig. 5H). Amino acids, peptides, and analogs showed trends analogous to those in the IM versus BR-stage comparison group; for example, the contents of l-cysteine, l-proline, l-tyrosine methyl ester, and other metabolites increased in the BR-stage versus RS fruit comparison group (Supplementary Data Table S14). Among the amino acids, peptides, and analogs, only l-alanyl-l-phenylalanine and *N*-acetyl-l-threonine decreased in abundance (Supplementary Data Table S14). Other metabolites, such as lauric acid, syringic acid, quinic acid, 3-indoleacrylic, and histamine, increased in abundance at this stage, and jasmonic acid, salicylic acid, vitamin B3, and syringic acid decreased in abundance (Supplementary Data Table S14). Moreover, KEGG annotations showed an association with α-linolenic acid metabolism and carbapenem biosynthesis (Supplementary Data Fig. S8D).

### Transcriptomic analysis

**Figure 6:**
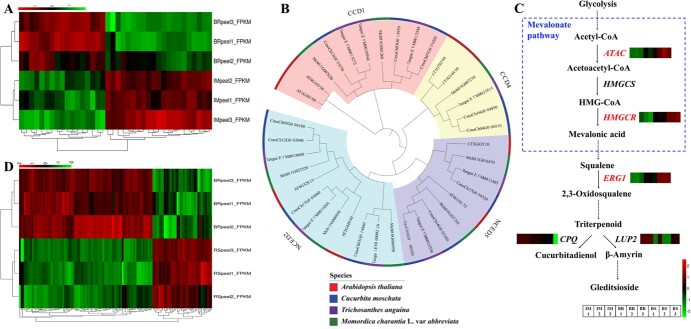
Transcriptome cluster heat maps of Mca fruit peel tissue at different stages and phylogenetic tree of the carotene cleavage dioxygenase (CCD) family and biosynthesis pathways of cucurbitadienol and gleditsioside. (A, D) Cluster heat maps of DEGs in the IM peel versus BR-stage peel comparison group (A) and the BR-stage peel versus RS peel comparison group (D). (B) CCD family phylogenetic tree comprising Mca, *T. anguina*, *C. moschata*, and *A. thaliana*. (C) Partial pathway of terpenoid backbone biosynthesis (ko00900) and sesquiterpenoid and triterpenoid biosynthesis (ko00909) for the production of cucurbitadienol and gleditsioside. Relative gene expression in the IM stage, BR stage, and RS is described in the boxes beside each gene; red color represents high relative expression and green color represents low relative expression.

According to the fruit peel transcriptome results, 515 differentially expressed genes (DEGs) were detected in the fruit at the early development period (IM stage compared with the BR stage); specifically, there were 254 upregulated genes and 261 downregulated genes (Supplementary Data Table S15). GO enrichment analysis of biological processes indicated that the enriched genes were involved in the auxin-activated signaling pathway, carbohydrate transport, the response to abscisic acid, and negative regulation of photosynthesis (Supplementary Data Fig. S9A). KEGG annotations indicated associations with cyanoamino acid metabolism, phenylpropanoid biosynthesis, plant hormone signal transduction, and fatty acid degradation pathways (Supplementary Data Fig. S9B). Among the 515 DEGs, the downregulated phytohormone signal transduction-related genes included genes encoding auxin-responsive proteins (*IAA4*/*16*/*25*/*29*, *SAUR50*), auxin-induced protein 15A (*AUX15A*), ethylene receptor 1 (*ETR1*), ethylene response sensor 1 (*ERS2*), ethylene-responsive transcription factor 2 (*ERF2*), and gibberellin 2-β-dioxygenase 8 (*GA2OX*), and the upregulated genes included those encoding ethylene-responsive transcription factor (*ERF071*/*CRF2*) and auxin-responsive protein SAUR50 (Supplementary Data Table S15). The expression of genes that encode proteins involved in polysaccharide biosynthesis and metabolism, such as sucrose-phosphate synthase 2 (*SPS2*), sucrose synthase (*SUS*), sugar transport protein 13 (*STP13*), fructose-bisphosphate aldolase 1 (*FBA1*) and β-fructofuranosidase insoluble isoenzyme CWINV1, was significantly increased (Supplementary Data Table S15). Increases in the expression of genes associated with changes in cell wall structure were also detected, including *β-galactosidase* (*GAL*), *expansin-A4* (*EXPA4*), and *pectinesterase 53* (*PME53*). Other upregulated genes, including *phenylalanine ammonia-lyase* (*PAL*), *peroxidase 51* (*POD51*), *1-aminocyclopropane-1-carboxylate oxidase 1* (*ACO1*), *alcohol dehydrogenase-like 7* (*ADH7*), *cinnamyl alcohol dehydrogenase 6* (*CAD6*), *carotenoid 9,10(9′,10′)-cleavage dioxygenase 1* (*CCD1*), and *β-carotene 3-hydroxylase 1* (*BCH1*), were found to play roles in Mca fruit growth (Fig. 6A, Supplementary Data Table S15).

In total, 4593 DEGs were detected, namely 1863 upregulated genes and 2730 downregulated genes, from the BR stage to the RS (Supplementary Data Table S16). GO enrichment analysis revealed that these genes were associated with integral components of membranes among the cellular component and calcium binding among the molecular functions (Supplementary Data Fig. S10A). KEGG annotations revealed associations with processes involving plant hormone signal transduction, carbon metabolism, starch and sucrose metabolism, glycerophospholipid metabolism, and biosynthesis of amino acids (Supplementary Data Fig. S10B). A phylogenetic tree based on the Mca, *Trichosanthes anguina*, *C. moschata*, and *Arabidopsis thaliana* carotene cleavage dioxygenase (CCD) families is shown in Fig. 6B. The expression of *9-cis-epoxycarotenoid dioxygenase NCED5* (*NCED5*) and *carotenoid cleavage dioxygenase 4* (*CCD4*) was downregulated and that of *NCED2* and *CCD1* was upregulated. Transcripts associated with cucurbitacin biosynthesis, such as *acetyl-CoA C-acetyltransferase* (*ACAT*), *hydroxymethylglutaryl-CoA reductase* (*HMGCR*), and *squalene monooxygenase* (*ERG1*), were upregulated in the BR-stage versus RS comparison group (Fig. 6C). Changes in the expression of other DEGs involved in phytohormone synthesis, perception and signaling, cell wall structure, and polysaccharide biosynthesis and metabolism are described in Fig. 6D and Supplementary Data Table S16.

### Combined transcriptome and metabolome analysis

**Figure 7:**
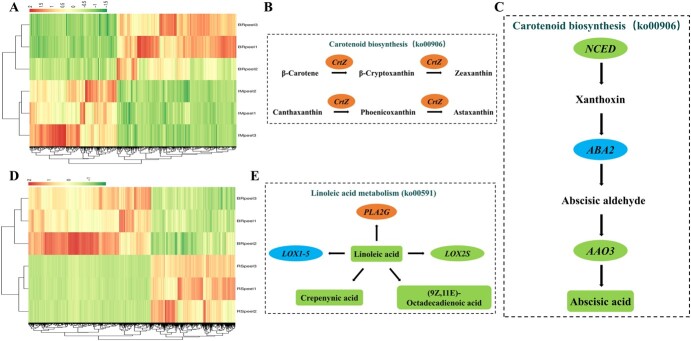
Heat maps and KEGG pathway enrichment diagrams of DEMs and DEGs. (A, D) Heat maps of DEMs and DEGs in the IM stage versus BR-stage comparison group (A) and BR-stage versus RS comparison group (D). (B) Partial pathway including the DEMs and DEGs involved in carotenoid biosynthesis (ko00906) in the IM-stage versus BR-stage comparison group. (C) Partial pathway including the DEMs and DEGs involved in carotenoid biosynthesis (ko00906) in the BR-stage versus RS comparison group. (E) Partial pathway including the DEMs and DEGs involved in linoleic acid metabolism (ko00591) in the BR-stage versus RS group. The DEGs are represented as ellipses (orange color indicates upregulated, green color indicates downregulated, and blue color indicates upregulated and downregulated), the content of DEMs in green decreased, and black words indicate no significant difference in content.

Coanalysis of DEMs and DEGs indicated the relationships between DEMs and DEGs, the results of which are presented as a heat map in Fig. 7A, together with network diagrams indicating the relationships between them. The results suggested that in the IM-stage versus BR-stage comparison group abscisic acid was positively regulated by *auxin-responsive protein* (*IAA4*/*14*), *auxin-induced protein 15A* (*AUX15A*), and *ethylene response sensor 1* (*ERS2*) and the TF *PIF7*, while *auxin-responsive protein SAUR50* played a negative role in abscisic acid biosynthesis (Supplementary Data Fig. S11A). According to the ko00270 KEGG map, aminotransferase *TAT2* and *homocysteine S-methyltransferase 3* played positive roles in l-methionine and pyruvic acid accumulation and negative roles in *S*-adenosylmethionine accumulation (Supplementary Data Fig. S11B), whereas the pyruvic acid content was increased by *1-aminocyclopropane-1-carboxylate synthase CMW33* (*ACS*). The content of l-glutamic acid was increased by *glutamine synthetase nodule isozyme* and *phosphoglycolate phosphatase 1B*, both of which were negatively correlated with pyruvic acid content in contrast to l-glutamic acid content. *Phosphoglycolate phosphatase 1B* and *oxalate–CoA ligase* were found to play different roles involving l-malic acid content, while *oxalate–CoA ligase* promoted the accumulation of oxalic acid (Supplementary Data Fig. S11C). In addition, β-carotene 3-hydroxylase (crtZ) positively affected β-carotene and canthaxanthin degradation, as indicated in Fig. 7B.

In the BR-stage versus RS comparison group, coenrichment annotations of DEMs and DEGs were carried out, the results of which showed involvement in α-linolenic acid metabolism, plant hormone signal transduction, and carotenoid biosynthesis pathways, according to the KEGG pathway information, and a heat map of these DEGs is shown in Fig. 7D. The plant hormone signal transduction pathway indicated that salicylic acid, jasmonic acid, and abscisic acid were positively correlated with the TFs *TGA9*/*bHLH35*/*MYC4* as well as *abscisic acid-insensitive 5* and negatively correlated with the abscisic acid receptor *PYL4* and the TF *MYBC1* (Supplementary Data Fig. S12A). In addition, salicylic acid and jasmonic acid were also positively regulated by jasmonic acid-amido synthetase *JAR1*, *auxin-responsive protein SAUR23*/*IAA25*/*27*/*29*, and the TFs *MYC2*/*bHLH93* and *ethylene response sensor 1* (*ERS1*); however, *indole-3-acetic acid-amido synthetase GH3.10* and *auxin-responsive protein IAA9*/*SAUR50* played negative roles in the accumulation of these metabolites (Supplementary Data Fig. S12A). In the linoleic acid metabolism pathway (Fig. 7E, Supplementary Data Fig. S10B), the accumulation trend of crepenynic acid was opposite that of *phospholipase A2-α* (*PLA2α*) but was the same as that of *linoleate 9S-lipoxygenase 5* (*LOX5*); *linoleate 13S-lipoxygenase 3-1* (*LOX3.1*) also played a positive role in crepenynic acid accumulation. Moreover, linoleic acid was positively correlated with *linoleate 13S-lipoxygenase 3-1* (*LOX3.1*) and negatively correlated with *lipoxygenase* (*LOX*) and *phospholipase A2* (*PLA2*). In the carotenoid biosynthesis pathway, abscisic acid was positively correlated with *9-cis-epoxycarotenoid dioxygenase* (*NCED*) and *abscisic-aldehyde oxidase* (*AAO2*) (Fig. 7F, Supplementary Data Fig. S10C).

## Discussion

Mca fruits are known for their taste and nutrient content, which are driven by diversified saponins, polypeptide polysaccharides, and other nutritionally functional components [31, 32], but molecular investigations on Mca fruits are scarce. Other studies involving bitter gourd genome assembly and domestication have revealed genetic maps of *Momordica* species; however, a high-quality genome has been less common in *Momordica* varieties and subspecies [2, 3, 21]. To provide more molecular information to aid research progress in Mca and other *Momordica* species, using a variety of sequencing platforms, we performed a genome assembly and comparative genome and genome-wide variation analysis and high-depth sequencing to construct the first Mca genome. This is a T2T gapless or nearly high-quality genome of a for 6 of the 11 chromosomes in *Momordica*. Other T2T genome assemblies have been completed for the human X chromosome and chromosome 8 [33, 34]. Naish *et al*. [35] studied the genetic and epigenetic landscape of *Arabidopsis* centromeres, and two gap-free reference genomes of rice are available [36]. In Cucurbitaceae species, Deng *et al*. [28] have assembled a gap-free watermelon genome of 11 chromosomes. These are fundamental for genome structure exploration and plant breeding as well as for trait selection. This 295.6-Mb genome was anchored to 11 chromosomes after Hi-C adjustment, which was slightly larger than genomes reported for bitter gourd [3, 21], silver-seed gourd [37], cucumber [12], zucchini [38], and pumpkin [18] but much smaller than those of other Cucurbitaceae, including snake gourd [23], wax gourd [22], sponge gourd [19, 20], chayote [26], watermelon [13, 14], melon [15–17], and bottle gourd [24, 25]. Our study provides additional genetic resources for bitter gourd genome assembly and for further evolutionary analysis of the Cucurbitaceae.

To better understand the evolutionary relationships between Mca and other members of the Cucurbitaceae, comparative genomic and genome-wide variation analyses were carried out. Figure 3B presents the divergence time of Mca and other species and shows that bitter gourd diverged from other Cucurbitaceae ~115–120 Mya. Cui *et al*. [2] reported that bitter gourd had diverged between 33.3 and 40.4 Mya, and Fu *et al*. [26] suggested that bitter gourd and other gourd species diverged 39–96 Mya; thus, bitter gourd diverged 33.3–40.4 Mya. According to our results (Fig. 3B), Mca diverged from bitter gourd 0.53–52.49 Mya; this is a large time span for divergence prediction owing to the quality of the bitter referenced gourd genome assembly. There is no fossil calibration information of varieties and original species, so we inferred that Mca diverged from bitter gourd 0.53–33.3 Mya, which is a relatively recent divergence event.

Through genome-wide variation analysis, we identified genes, such as *gibberellin 2-beta-dioxygenase 8* (*GA2OX8*) and *polygalacturonase* (*PG*) (Table 4), that have common InDels and SNPs and may participate in Mca fruit ripening. In *A. thaliana*, *gibberellin 2-beta-dioxygenase 7* (*GA2OX7*) participates in gibberellin (GA) synthesis and indole-3-acetic acid conjugation with amino acids [39, 40]. *Polygalacturonase* (*PG*) is involved in cell wall metabolism, acting in concert with pectinesterase in the ripening process [41, 42]. These genes may be related to the phytohormone synthesis and fruit texture changes that occur during ripening of Mca fruit, all of which may contribute to the unique Mca characteristics. Other genes, such as *terpene synthase 10* (*TPS10*) and *flavonol synthase* (*FLS*), involving SVs were also predicted; these genes may also play a role in Mca fruit quality. *Terpene synthase 10* (*TPS10*) was demonstrated to be involved in monoterpene biosynthesis [43], and Cucurbitaceae family members are rich in terpenes, which are attractants with antioxidant, anti-inflammatory, and other nutritional values [44]. *Flavonol synthase* (*FLS*) is involved in the biosynthesis of flavonol and kaempferol, which are associated with fruit pigment and have antioxidant characteristics [45, 46]. The absence of these SVs and presence–absence variations (PAVs) in genes may have been essential for the formation of the unique Mca features during its divergence from bitter gourd.

**Figure 8:**
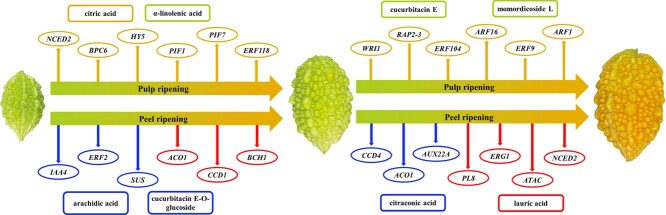
Schematic showing changes in the accumulation of metabolites in Mca peel and pulp tissues during ripening. From left to right: fruit at the IM stage, BR stage, and RS. The upper section represents the pulp ripening process. ATAC-seq genes are shown in yellow ellipses, and metabolites are shown in boxes (metabolites whose abundance increased are shown in yellow and metabolites whose abundance decreased in shown in green). The lower section represents the peel ripening process, with transcripts shown in ellipses (blue arrows indicate decreased levels and red arrows indicate increased levels) and metabolites shown in boxes (metabolites whose abundance increased are shown in red and metabolites whose abundance decreased are shown in blue).

Throughout the Mca fruit pulp ripening process, the DEMs in pulp samples mainly include amino acids, lipids, organic acids and other substances, such as saponins and cucurbitacin. Saponins were verified as being the main bioactive substances with antihyperglycemic activities in *M. charantia* [47]. The content of isovitexin-7-*O*-glucoside (saponarin) decreased in both comparison groups. Substances unique to Cucurbitaceae, such as cucurbitacin E, candicine, cucurbitacin D-*O*-glucoside, cucurbitacin E-*O*-glucoside, momordicoside F2, momordicine IV, and momordicoside L, differentially accumulated in the postripening stage (BR-stage versus RS). Cui *et al*. [2] reported that the main cucurbitane triterpenoid synthesized in bitter gourd is cucurbitacin E, which is the main bitter substance in watermelon [48] and is converted from cucurbitacin I through the action of *ClACT* and *ClBi* [49–51]. Momordicine and vitamins with antioxidant activity are abundant in bitter gourd [52–54]. These substances play important roles in the medicinal value of Mca fruit. The following DEMs were also detected: betaine; l-methionine; l-cystine; l-tyrosine methyl ester; γ-linolenic acid*; α-linolenic acid*; malonic acid; citric acid*; and plant hormones such as jasmonic acid, salicylic acid, and abscisic acid. However, the changes in the expression of DEGs were consistent with the increased accumulation of these metabolites (Supplementary Data Tables S10 and S11). As a previous study described, a number of amino acids were associated with fruit flavor and aroma; these included leucine, tyrosine, and phenylalanine, which are related to branched-chain esters and aromatic esters [55, 56]. These metabolites and genes allow a better understanding of the regulatory and biochemical variations in Mca fruit composition during ripening (Fig. 8). The DEMs in the Mca peel were different from those in the fruit pulp, which suggested that studies on the differential accumulation of these and the uses of peel and pulp tissues are worthy of further research.

During the Mca fruit peel ripening process, the peel color changes from green to yellow/orange at maturity, so DEGs involved in fruit chlorophyll degradation and carotenoid pigment accumulation are expected to play crucial roles in the Mca fruit peel color change. In the comparison of the BR stage with the RS, DEGs such as *9-cis-epoxycarotenoid dioxygenase NCED* (*NCED2*/*5*), *carotenoid 9,10(9′,10′)-cleavage dioxygenase 1* (*CCD1*), and *carotenoid cleavage dioxygenase 4* (*CCD4*) were detected (Supplementary Data Table S14) and were classified as belonging to the CCD family, whose members are related to fruit pigment accumulation [57]. According to a recent Cucurbitaceae study, *NCED2* showed much higher expression during the snake gourd peel RS, during which time the color changed from green/white to red at maturity [23]. Changes in *CCD4* gene expression are also involved in squash, pumpkin, and watermelon pulp ripening [58–60]. Furthermore, the accumulation of high amounts of β-carotene also occurs during bitter gourd fruit ripening [61]. The biosynthesis and metabolism of cucurbitacin, a metabolite unique to Cucurbitaceae, are important for the study of Mca fruit features. According to our results (Figs 6C and 8, Supplementary Data Table S14), genes promoting the synthesis of cucurbitacin, including *acetyl-CoA C-acetyltransferase* (*ACAT*), *hydroxymethylglutaryl-CoA reductase* (*HMGCR*) and *squalene monooxygenase* (*ERG1*), were upregulated in the BR-stage versus RS comparison group (Figs 6C and 8). Previous studies of the cucurbitacin biosynthesis pathway showed that in melon fruit *Cm890* and *Cm180* were annotated in the synthesis of cucurbitacin D and B [62]. Shang *et al*. [63] described that cucurbitacin C was derived from *Cs540* and *Cs160*, and cucurbitacin I and E are reportedly synthesized through *Cl890A*/*Cl890B* and *Cl180* [49]. However, specific names for the genes involved in the synthesis of cucurbitacin have not been defined. Further study of the roles of these genes in Mca is needed. DEGs associated with Mca fruit enlargement, including auxin-, ethylene-, GA-, abscisic acid- and pectin biosynthesis-related genes, were also identified (Supplementary Data Tables S10 and S11). Genes involved in cell wall modification, such as the members of the expansin family, *pectate lyase* (*PL*), *pectinesterase* (*PME*), *GAL*, *PG*, and *cellulose synthase-like protein* [64], during the fruit ripening process have also been found in melon, snake gourd, and chayote [17, 23, 26]. Further analysis of the transcriptome changes should allow a more complete understanding of the regulatory and biochemical changes that occur during Mca fruit ripening and that determine fruit quality and nutritional value.

## Materials and methods

### Genome sequencing and assembly

Fresh and healthy Mca plants for genome sequencing were collected in Wuzhong district, Suzhou, Jiangsu Province; these plants are generally named ‘Jin ling zi’ or ‘Lai pu tao’ as a variety of bitter melon. After harvesting, samples were immediately frozen in liquid nitrogen and preserved at −80°C for DNA extraction. By the use of the modified cetyl-trimethylammonium bromide (CTAB) method [65], high-quality genomic DNA was extracted from Mca leaves. Redundant RNA was removed using RNase A, and agarose gel electrophoresis was used to check the quality of the DNA. For Illumina sequencing, a short-read (350 bp) library was constructed and sequenced on an Illumina NovaSeq platform to obtain clean reads, which were then used to assess the genome size, GC content, and heterozygosity. For PacBio sequencing, a long-read library was constructed from genomic DNA, which was fragmented to 15 kb according to the manufacturer’s instructions. Then, this library was sequenced on a PacBio Sequel II platform. Low-quality reads and sequence adapters were removed to obtain clean subreads. Hifiasm software (v0.14) [66] was used to splice high-precision CCS data to obtain genomic sequences. The assembly results were evaluated using BWA-MEM [67], CEGMA v2.5 (default settings) [68], and BUSCO v5 [69].

To advance the quality of genome assembly, we used Hi-C technology to help anchor contigs. Based on sequencing-by-synthesis (SBS) technology, an Illumina high-flow sequencing platform was employed to sequence the Hi-C database to obtain raw data. Then, high-quality clean data were obtained by filtering the raw Hi-C sequencing data and low-quality reads. Clean read pairs were obtained from the Hi-C library and were subsequently mapped to the originally assembled Mca genome by BWA (bwa-0.7.17), with the default parameters. Contigs mapping to the matching paired reads were used for the performance of Hi-C-associated scaffolding. Null reads, including those near start sites, artifacts from PCR amplification, those resulting from random breaks, those corresponding to large fragments, those corresponding to small fragments, and those corresponding to extreme fragments, were filtered and removed. Then, we used Lachesis [70] with the agglomerative hierarchical clustering method to successfully cluster contigs into chromosomes. Lachesis was further applied to order and orient the clustered contigs. The specific parameters used were as follows: cluster_MIN_RE_SITES = 24; CLUSTER_MAX_LINK_DENSITY = 2; ORDER_MIN_N_RES_IN_TRUNK = 6; and ORDER_MIN_N_RES_IN_Shreds = 6. The contigs were then manually mapped and evaluated to obtain genome sequences at the chromosome level.

### Genome annotation

TEs and tandem repeats were annotated via the following workflows. First, homology-based and *de novo* approaches were used to analyze TEs. We then built a *de novo* repeat genome library by the use of RepeatModeler, programs can automatically estimate repeat finding, RECON (v1.08) [71] and RepeatScout [72]. Then, full-length long terminal repeat retrotransposons (FL-LTR-RTs) were identified using both LTRharvest (−minlenltr 100 -maxlenltr 40 000 -mintsd 4 -maxtsd 6 -motif TGCA -motifmis 1 -similar 85 -vic 10 -seed 20 -seqids yes) and LTR_finder (-D 40000 -d 100 -L 9000 -l 50 -p 20 -C -M 0.9). The high-quality intact FL-LTR-RTs and non-redundant LTR library were then produced by LTR_retriever. A non-redundant species-specific TE library was subsequently built by merging the TE sequence library above with the known Repbase (v19.06), REXdb (v3.0), and Dfam (v3.2) databases. Final TE sequences identified and classified by homology searches against the library using RepeatMasker v4.10, and tandem repeats were annotated by the Tandem Repeats Finder and MIcroSAtellite identification tool (MISA v2.1).

Gene predictions and annotations were integrated in three ways, namely *de novo* prediction, homology searches, and transcript-based assembly, to annotate the protein-coding genes in the genome. Two *ab initio* gene-prediction software tools, namely Augustus (v2.4) and SNAP (2006-07-28), were used for the prediction of *de novo* gene models. For the homolog-based approach, GeMoMa (v1.7) software was used, in which references gene models from other species were used. For transcript-based predictions, RNA sequencing data were mapped to the reference genome using HISAT (v2.0.4) and assembled by StringTie (v1.2.3). GeneMarkS-T (v5.1) and PASA (v2.0.2) software tool was then used to predict genes based on the assembled transcripts and unigenes (and full-length transcripts from PacBio (ONT) sequencing), respectively, and assembly was performed using Trinity (v2.11). The gene models from prediction were integrated using EVM software (v1.1.1) and corrected by PASA. The final gene models were annotated by searching the GenBank, NR (20200921), TrEMBL (202005) [73], Pfam (33.1), SwissProt (202005), KOG (20110125), GO (20200615), and KEGG (20191220) [74] databases.

Pseudogenes usually have sequences similar to those of functional genes but may have lost their biological function because of genetic mutations, such as InDels. The GenBlastA (v1.0.4) program was used to browse the genomes after the hiding of functional gene prediction. Supposed candidates were then identified by searching for premature stop codon and frameshift mutations using GeneWise (v2.4.1). Non-coding RNAs are usually divided into several groups, including miRNAs, rRNAs, tRNAs, snoRNAs, and snRNAs. tRNAscan-SE (v1.3.1) was used to predict tRNAs, with criteria specific for eukaryotes. Barrnap (v0.9) was applied to identify rRNA genes. miRNAs were picked out by searching the miRBase (release 21) database, and snoRNA and snRNA genes were analyzed using INFERNAL against the Rfam (release 12.0) database (BMK Corporation).

### Telomere detection and karyotype analysis

We detected the telomeres of the Mca chromosomes according to the methods of Song *et al*. [36]. Then, genomic karyotype identification and distribution of important repeats by fluorescence *in situ* hybridization were carried out. For this, the root tips of Mca samples with a length of 2–3 cm were placed in a 0.5-ml centrifuge tube with holes at the top for dye preparation. First, the centrifuge tube containing the root tips was placed in an inflatable tank filled with 0.9–1.0 MPa nitrous oxide for 2 hours. In an ice bath, 90% precooled glacial acetic acid was used to fix the split phase, which was then washed twice with ddH_2_O for subsequent experiments. After enzymolysis, fragmentation, and dropping, the chromosome samples were examined under an ordinary optical microscope or phase-contrast microscope to find target cells in the middle of mitosis, which were saved for future use. The probe was labeled by the nick translation method, and the fully reactive labeled probe was purified with a Qiagen kit. The purified probe was stored at −20°C until use. The telomeres were labeled with pTa794 and pTa71 plasmid DNA via synthetic terminal (TTTAGGG)_6_ probes and using 5S rDNA and 18S rDNA probes, respectively. Then, 8 μl of TE2 × SSC (pH 7.0) solution, 0.25 μl of probe solution, and two different fluorescent color-labeled probes were added to each slide. After adding the labeled probe solution to each slide, a cover-slip was added. The slides were subsequently heated at 80°C for denaturation for 5 minutes, after which they were placed in a hybridization box. The materials in the hybridization box were subsequently allowed to hybridized overnight at 37–42°C. Then, each slide was washed with 2× SSC solution two or three times at room temperature and then dried under a stream of air. Then, 8 μl of 4′,6-diamidino-2-phenylindole (DAPI) dissolved in anti-fluorescence quencher solution was applied, and a cover-glass was then added. The slides whose materials were subjected to *in situ* hybridization were imaged with a charge-coupled device a high-resolution fluorescence microscope for accurate karyotype analysis (OMIX Technologies Corporation).

### Genome-wide variation analysis

To investigate the genomic differences between Mca and bitter gourd, we performed a genome-wide variation analysis. Taking the assembled Mca as the reference genome, we used the complete bitter gourd assembly genome published by Matsumura *et al*. [3] for alignment analysis. MUMmer v4.0 [75] with the parameters -c 500 -b 500 -l 100 —maxmatch was used for genome-wide alignment, and the raw alignment results were further filtered using a delta filter, with the parameters −1 -i 90 -l 500. Then, SyRI [76] with the default parameters was used to detect the SVs among the filtered delta files. For the genes where mutations were detected, clusterProfiler v3.14.0 was used, and GO and KEGG enrichment analyses and annotations were performed. ANNOVAR was performed as part of a software program [77] used to determine the functional effects (BMK Corporation).

### Comparative genomic analysis methods

OrthoFinder v2.4 software [78] was applied to organize the protein sequences of 15 species into families (the diamond alignment method was adopted, and the alignment e value was 0.001), and the obtained gene families were annotated using the Panther v15 database [79]. Finally, the unique gene families of these species were determined by GO and KEGG enrichment analyses. The gene copy number of each gene family in each species was analyzed, and the unique gene families of these species were analyzed using clusterProfiler v3.14.0 [80] for GO and KEGG enrichment analysis. Using single-copy gene sequences, IQ-TREE v1.6.11 [81] was used to build an evolutionary tree. Specifically, MAFFT v7.205 [82] (parameter -localpair —maximal 1000) was applied to contrast the sequence of every single-copy gene family, and then the regions with poor sequence alignment or large divergences were filtered out using gBlocks v0.91b [83] (parameter -B5 = h). Finally, all the well-aligned gene family sequences of each species were attached end to end to gain supergenes, after which ModelFinder [84], a model detection tool in IQ-TREE, was used. The most excellent model was the JTT + F + I + G4 one. Then, using this most excellent model, we constructed an evolutionary tree by the maximum likelihood method, setting the bootstraps as 1000. The divergence time was calculated by the PAML v4.9i software package MCMCTree 9i [85]. Computational Analysis of gene Family Evolution (CAFÉ; v4.2) software was applied to predict the gene families that contracted and expanded in Mca species relative to its ancestors. Forward selection analysis was carried out with the CodeML module in PAML. The specific method of collinearity analysis involved the use of DIAMOND v0.9.29.130 [86] to identify the similar gene pairs between two species (e < 1E^− 5^, C score >0.5, where the C score value is screened by JCVI software). Then, according to the gff3 file, we determined whether similar gene pairs were adjacent on a chromosome. This process was mainly carried out through MCScanX [87] (parameter -m5). Finally, the genes in all collinear blocks were obtained. The *K*_s_ and 4DTv combination method is currently widely used to identify WGD events. WGD v1.1.1 software [88] and a custom script (https://github.com/JinfengChen/Scripts) were used to identify WGD events within Mca. For this, LTR transposons were the focus of attention, as they typically have been. We used LTR_ FINDER v1.07 software [89] to search for LTR sequences with scores ≥6 in the genome (i.e. via parameter -S6) and filter LTRs corresponding to duplicate results at the same time. After MAFFT comparisons and Kimura model-based distances were calculated in EMBOSS v6.6.0, the LTR insertion times were analyzed [90] (BMK Corporation).

### ATAC-sequencing analysis

Fresh fruit samples at three different maturity periods, namely IM fruit (14 days after flowering), BR-stage fruit (28 days after flowering), and RS fruit (35 days after flowering), were collected in Wuzhong district, Suzhou, Jiangsu Province. Three biological replicates in each group and Mca fruit pulp samples were used for ATAC-seq. The sample preparation and sequencing procedures were processed using the common steps described by Illumina Corporation. The raw reads contained adapters (with length <35 bp) and low-quality reads (reads with >10% N, and reads with a base quality value Q ≤ 10 proportion of >50% of the whole read), which were both cleared by Cutadapt software. Then, the cleared high-quality reads given in FASTQ arrangement were used for further analysis. Bowtie2 software was used to contrast the clean reads gained from the sequencing of every sample with the reference genome to obtain the alignment efficiency of the sample reads and the positional information of the reads on the genome. DeepTools v2.07 was applied to outline the density distribution of the sequences. The 3-kb interval upstream and downstream of the transcription start site (TSS) of every gene was read, and represented as heat maps. The read abundance throughout the whole genome was statistically analyzed by the sliding window method, with 10 kb taken as the unit length of interval and the chromosomes divided into multiple small windows. The mapped reads that landed in each window were counted as read abundance, and the Pearson correlation coefficient of the normalized read abundance was calculated across all the samples. A sample correlation clustering heat map was made. MACS2 v2.1.1 software was used to perform peak extraction. The DiffBind package was used to analyze differences in peaks in different comparison groups. Specifically, the number of read counts supported by peaks in each sample was calculated, and the affinity score was calculated based on the number of counts (i.e. the standardized read); the affinity score was used as the input of DESeq2 [91] software for differential screening of samples in each group. The screening criteria were as follows: FC >1.5 and *P* value <.05. Here, FC is defined as the read count multiple of the treatment group relative to the control group, i.e. the treatment group/control group. Motif analysis was performed on the basis of the difference peak analysis results, and MEME-ChIP software was also used to identify and annotate the motifs. Tomtom was used to align the detected motif sequences with known motifs (BMK Corporation).

### Metabolome analysis

Mca fruit pulp and peel samples at the three maturity periods were used for metabolome analysis, and three biological replicates were included in each group. The samples were subjected to vacuum freeze-drying and HPLC–MS analysis. Based on the self-built database, the materials were characterized according to their second-order spectral information and then quantified by a multireaction monitoring (MRM) model. Standardized processing data were used for further analysis. The overall metabolic differences and variability of each group of samples were preliminarily determined with a principal component analysis of the samples. The square of the Spearman rank correlation coefficient *R* (Spearman rank correlation) was used as an evaluation index of biological repetition correlation. The detected metabolites were contrasted for classification and pathway information in the KEGG, HMDB, and LipidMaps databases. According to the grouping information, the difference multiples were calculated and compared, and a *t*-test was applied to determine the difference significance (*P* value) of every metabolite. The R language package ropls was applied to represent orthogonal projections to latent structures discriminant analysis (OPLS)-DA modeling, and 200 permutation tests were carried out to verify the reliability of the model. The variable importance in projection (VIP) value of the model was calculated using multiple cross-validation. The method of composing the difference multiple, the *P* value and the VIP value of the OPLS-DA model was adopted to check the DEMs. The checking criteria were FC >2, *P* value <.05 and VIP >1. The significance of the DEMs associated with KEGG pathway enrichment was determined using a hypergeometric distribution test (BMK Corporation).

### Transcriptome analysis

Mca fruit peel samples from three maturity periods were used for transcriptome analysis, and three biological replicates were included for each group. The original data were obtained by sequencing on the Illumina platform, and clean data were obtained after filtering. Mapping data were obtained by sequence alignment with the specified reference genome, and then the quality of the database was evaluated. According to the gene expression of different sample groups, the DEGs were annotated and enriched. DESeq2 (v1.6.3, default value: test = ‘Wald’, fittype = ‘parameter’) [91] was used to analyze the differences in expression between sample groups. When detecting DEGs, we used a screening criterion of FC ≥2 and false discovery rate <.01. The GO and KEGG databases were used to annotate and determine the enriched DEGs.

### Combined transcriptome and metabolome analysis

According to the DEM analysis results of this experiment, combined with the transcriptome analysis results of the DEGs, the differentially expressed genes and differential metabolites in the same group were annotated to the KEGG pathway map at the same time, the results of which can help to perform communications between genes and metabolites. Selecting genes and metabolic pathways with a *P* value <.05 for priority analysis can save time for screening data and quickly find the pathways related to the research purpose for follow-up analysis. For this, we grouped the genes according to each difference, calculated the correlation coefficients between all genes and metabolites based on the Pearson correlation method, preprocessed the data with the *Z*-value transformation method before calculating the correlations, and then screened them according to the correlation coefficient (CC) and corresponding *P* value. The screening threshold criteria were as follows: | CC | > .80 and CCP < .05 (BMK Corporation).

## Supplementary Material

Web_Material_uhac228

## Data Availability

The *M. charantia* L. var. *abbreviata* Ser. (Mca) raw genome sequencing, assembly data and TE annotations are available from the NCBI under project ID PRJNA873243.
