# Supplementary material for: Telomere-to-telomere genome assembly of bitter melon (Momordica charantia L. var. abbreviata Ser.) reveals fruit development, composition and ripening genetic characteristics
Source: Hortic Res. 2022 Oct 11;10(1):uhac228. doi: 10.1093/hr/uhac228 (PMC9832870; doi:10.1093/hr/uhac228)
Supplement: Web_Material_uhac228 [file web_material_uhac228.zip › Supplemental files/Supplemental Figures.docx]

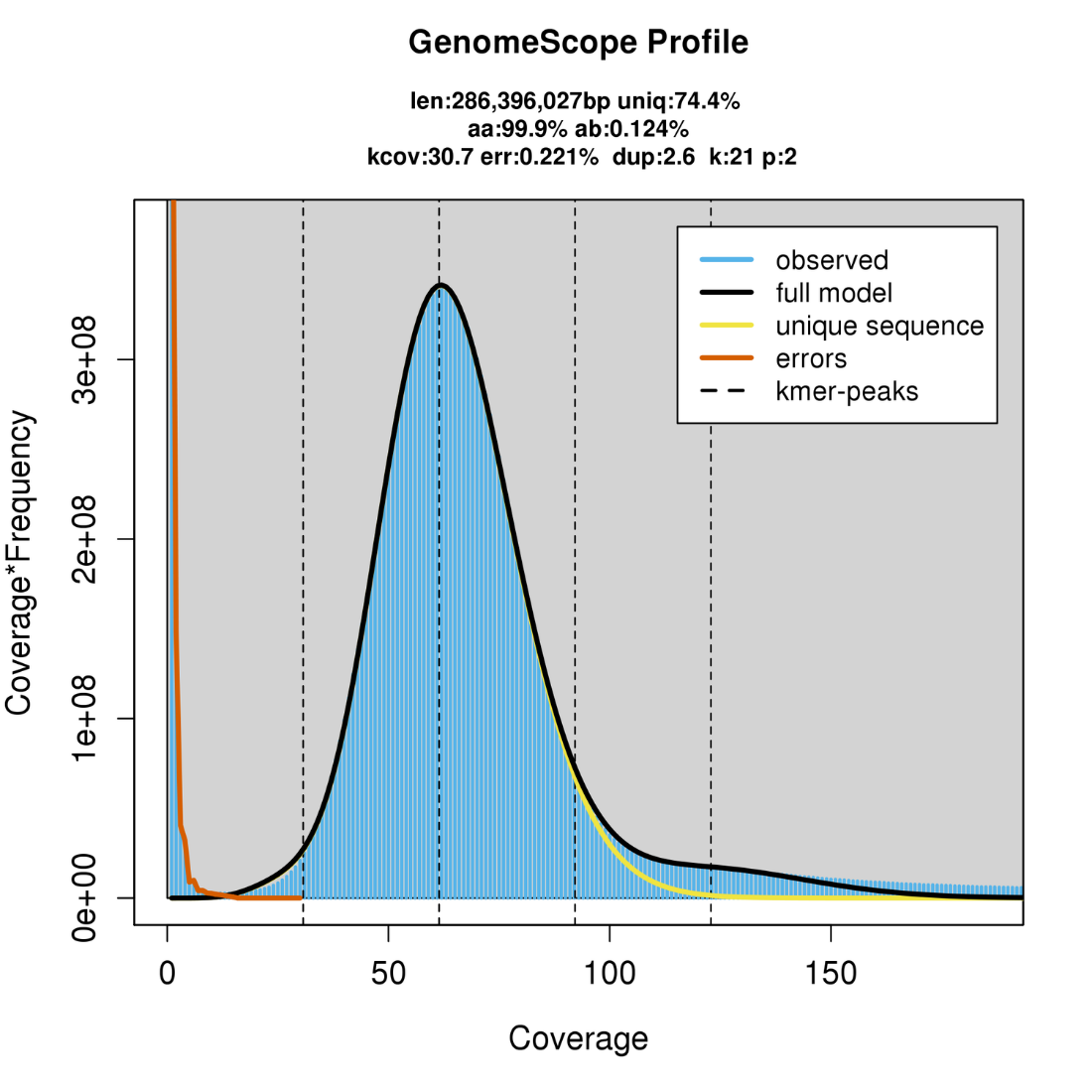


**Figure S1 Kmer distribution map.**


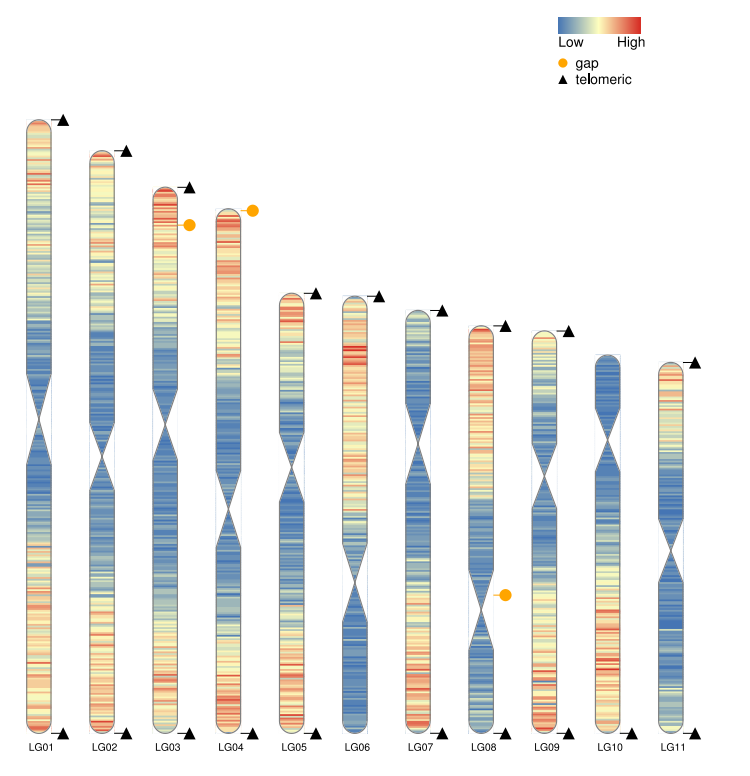


**Figure S2 Distribution map of telomeres and centromeres on Mca 11 chromosomes.**


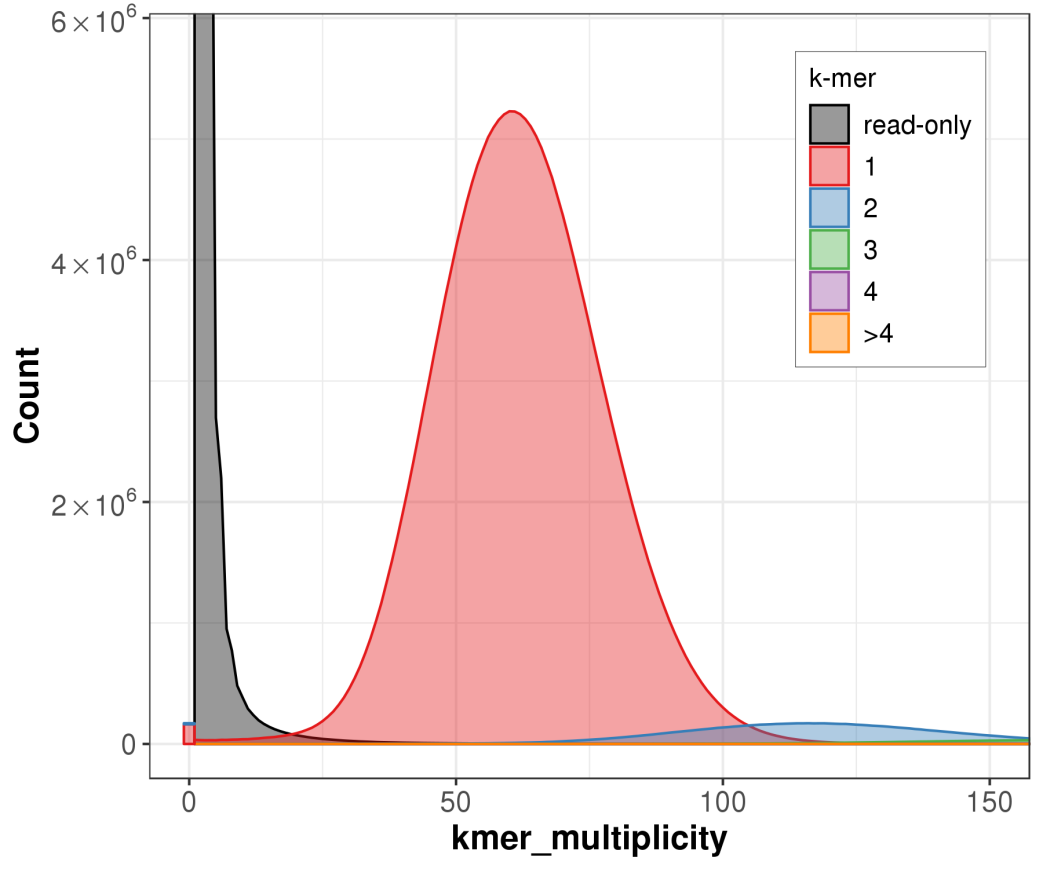


**Figure S3 Evaluation of kmer_multiplicity**


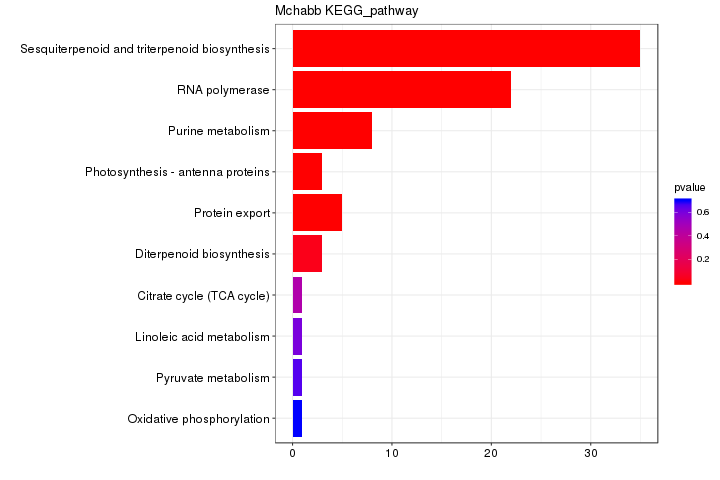


**Figure S4 KEGG enrichment analysis of Mca specific gene families.**

**A**


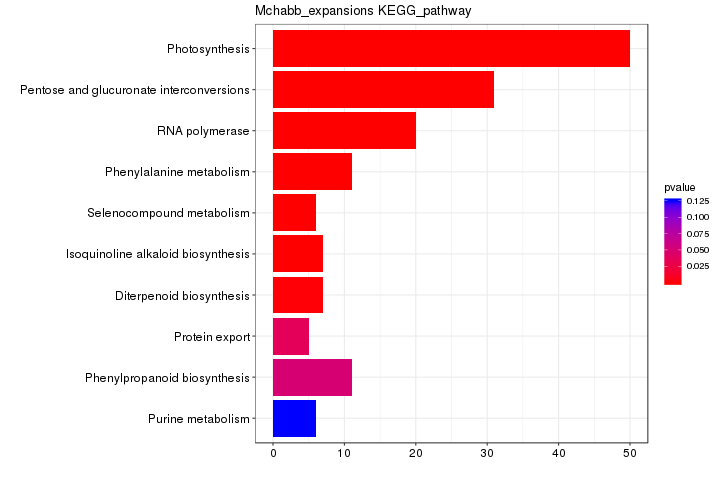


**B**


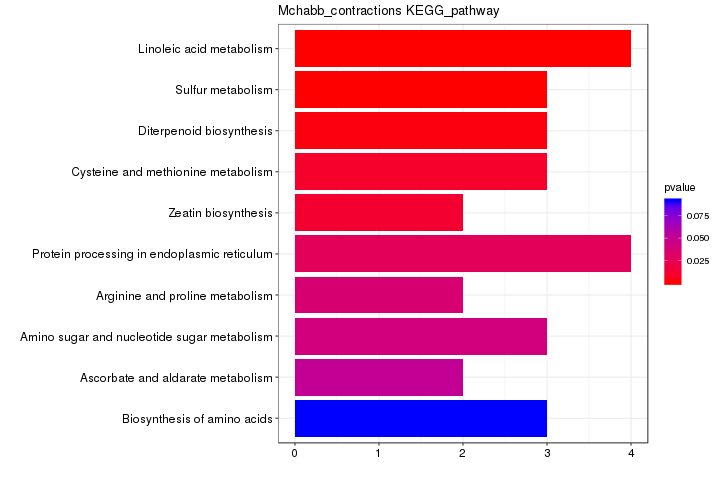


**Figure S5 KEGG enrichment analysis of expanded genes (A) and contracted genes (B).**

**A**


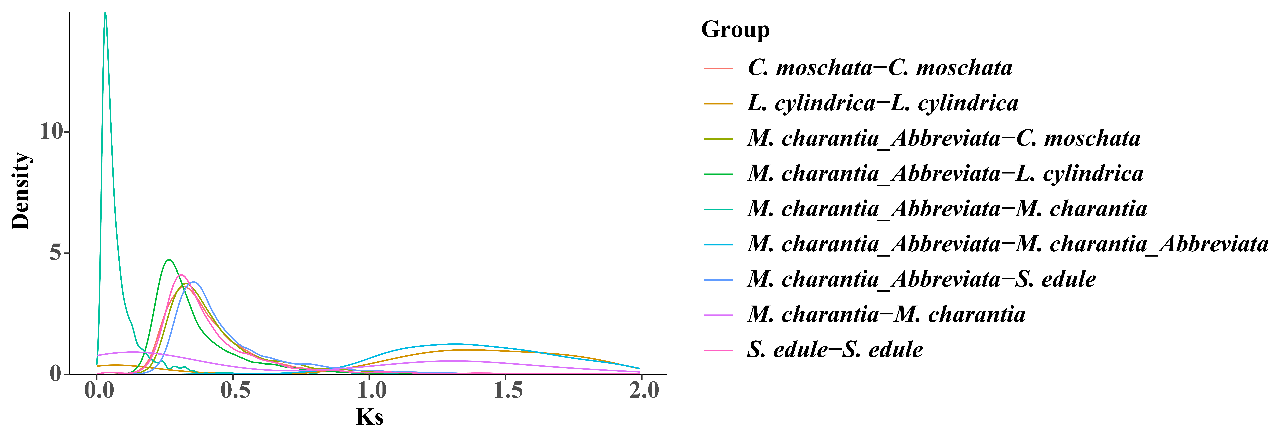


**B**


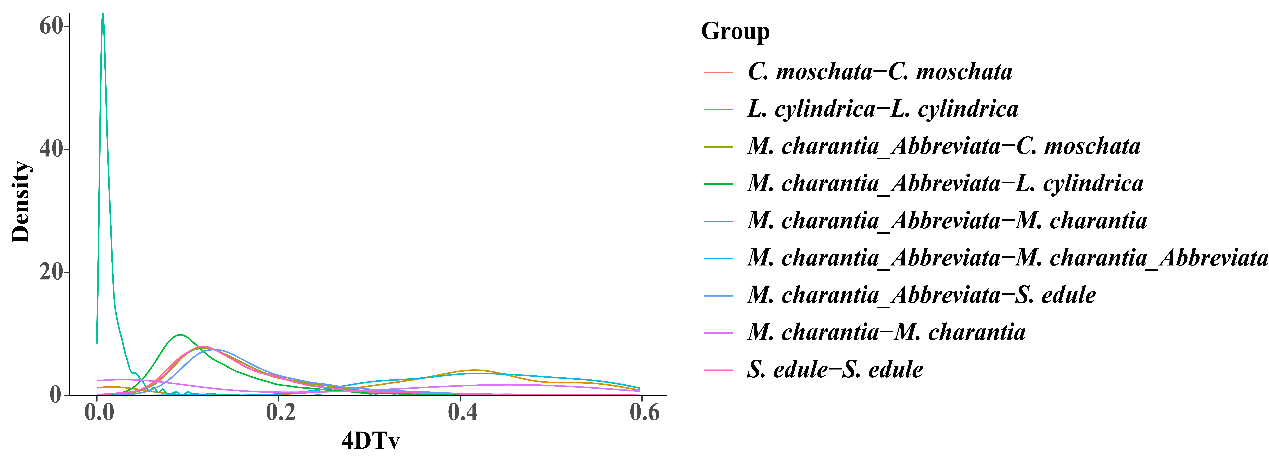


**Figure S6 Distribution map of Ks (A) and 4DTv (B).**

**A**


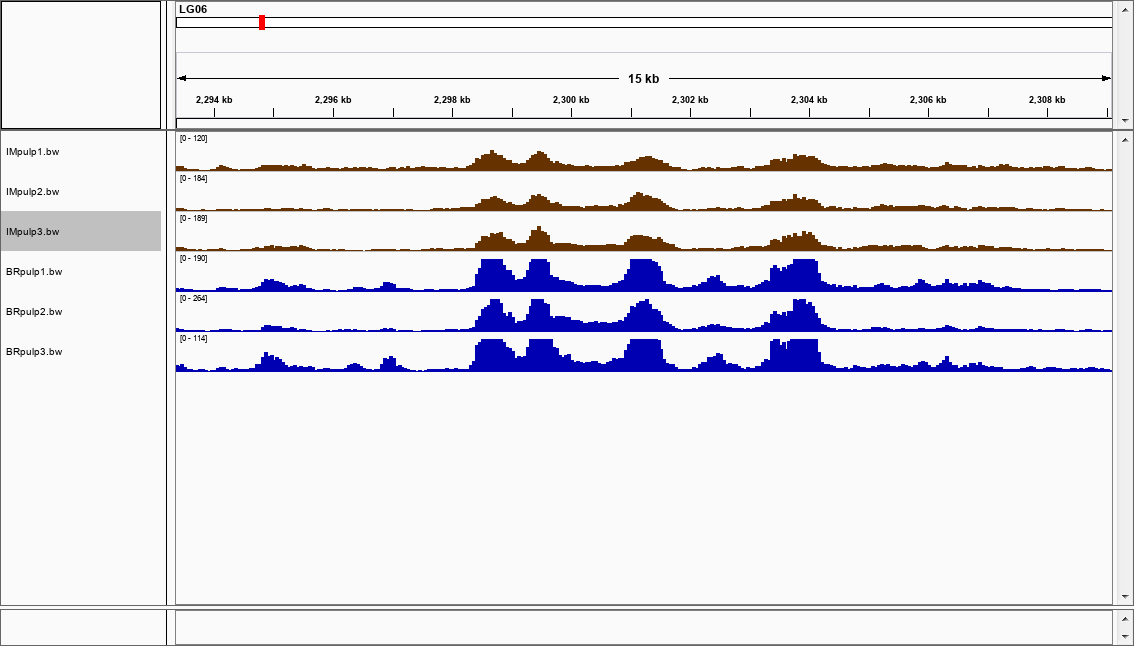


**B**


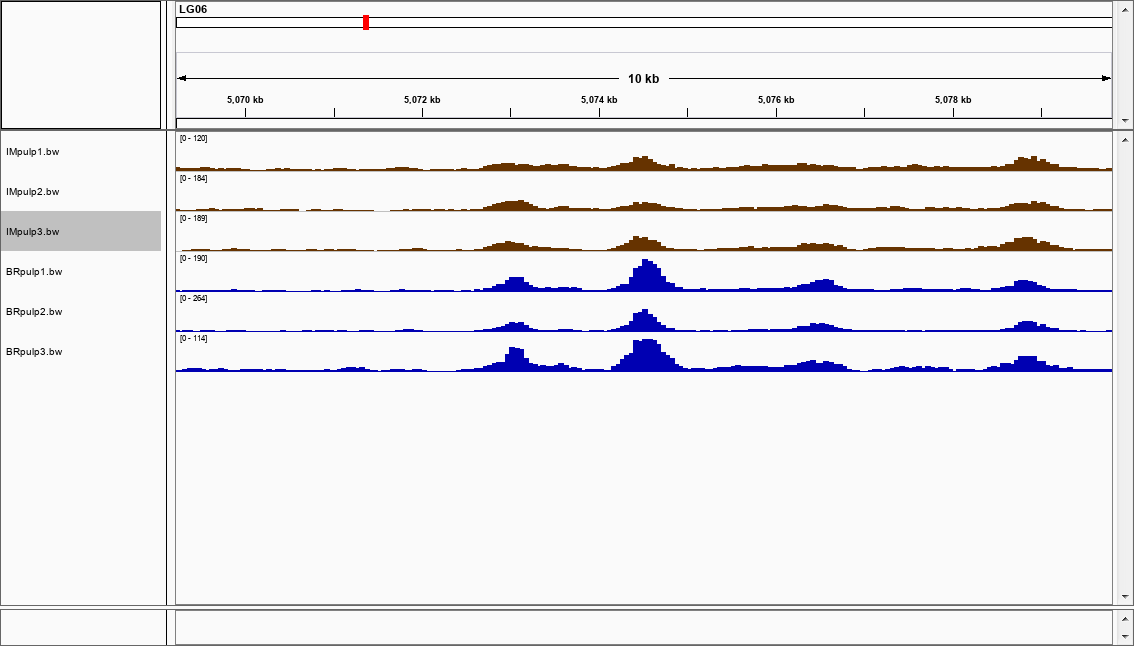


**C**


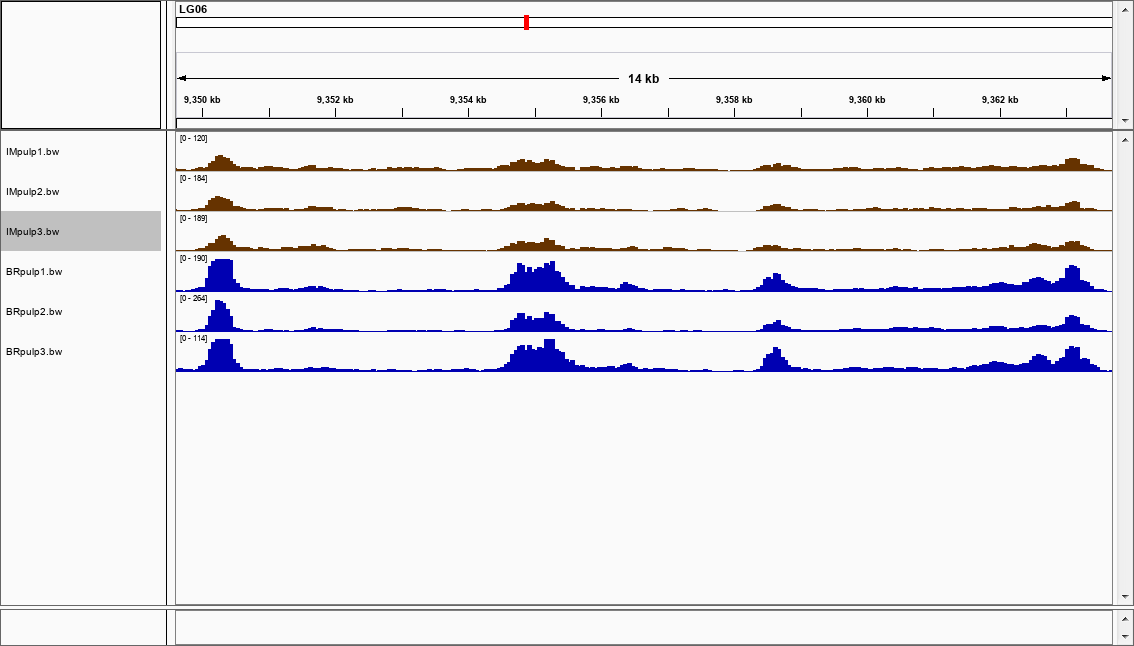


**D**


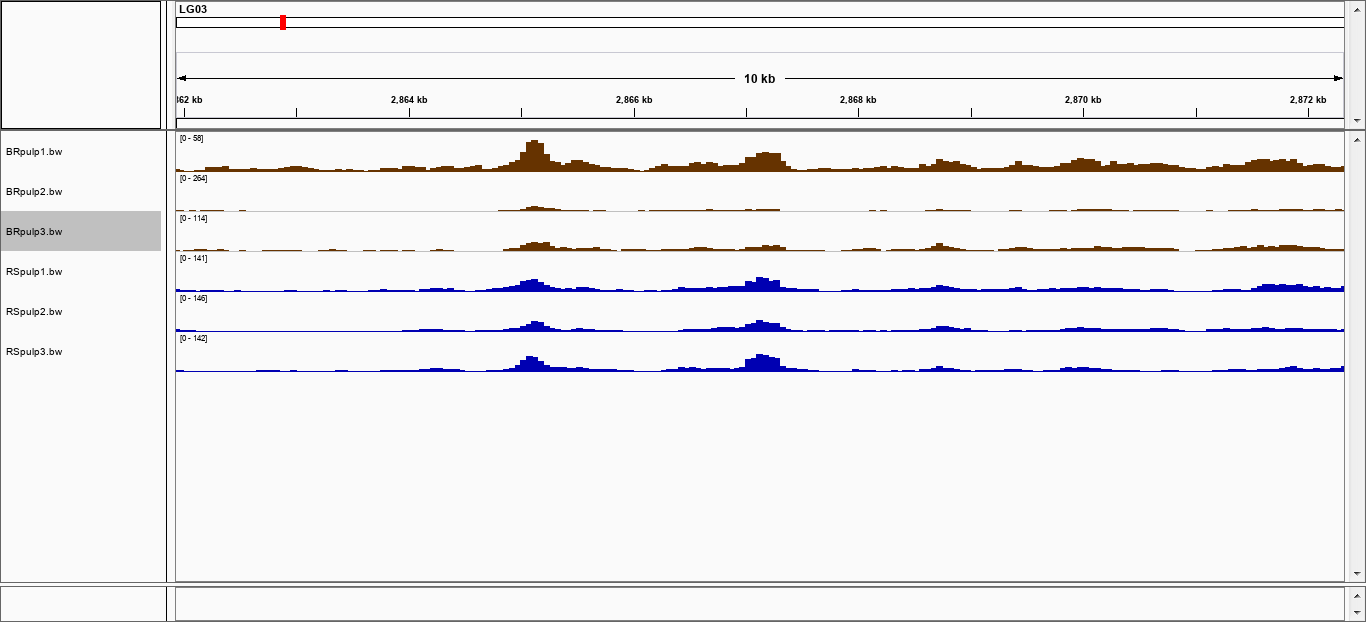


**E**


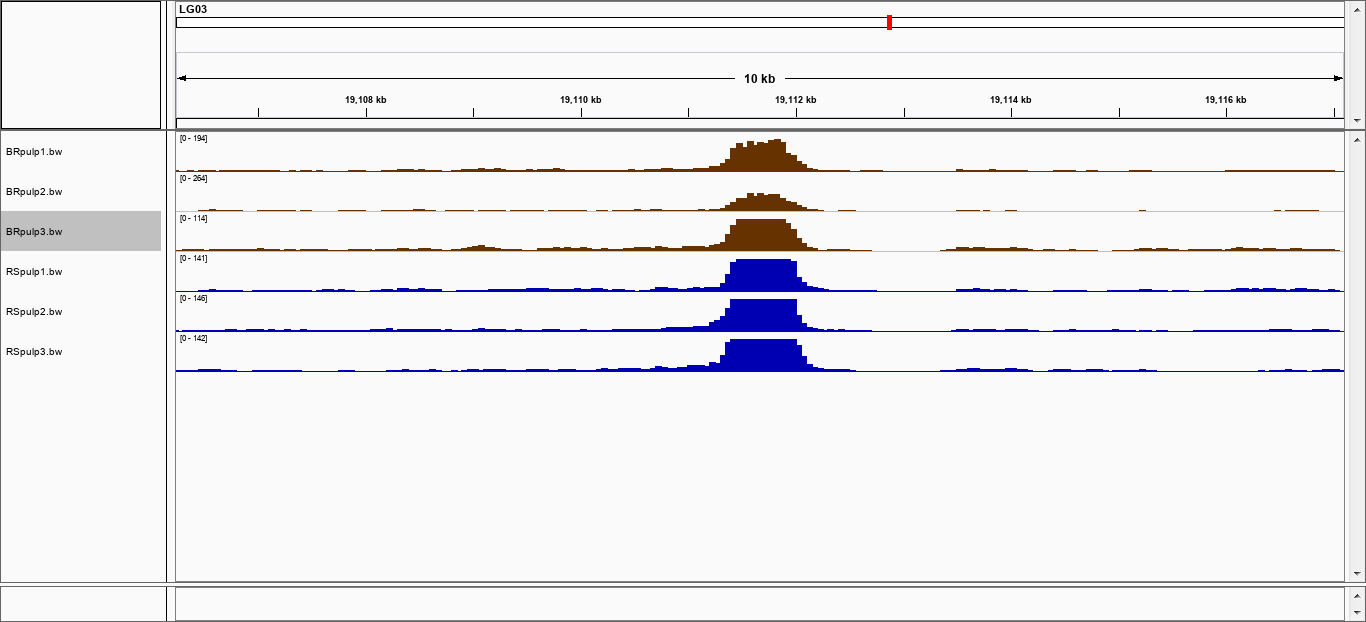


**Figure S7 IGV analysis of ERF4 (A), ERF60(B), ARF18 (C) with matched TFs in IM vs BR group and ERF118 (D), ARF1 (E) with matched TFs in BR vs RS group.** The X-axis is different for sample groups, Y-axis is part of chromosome, red lines are matched TFs; D IGV analysis of, the X-axis is different for sample groups, Y-axis is part of chromosome, red lines are matched TFs.


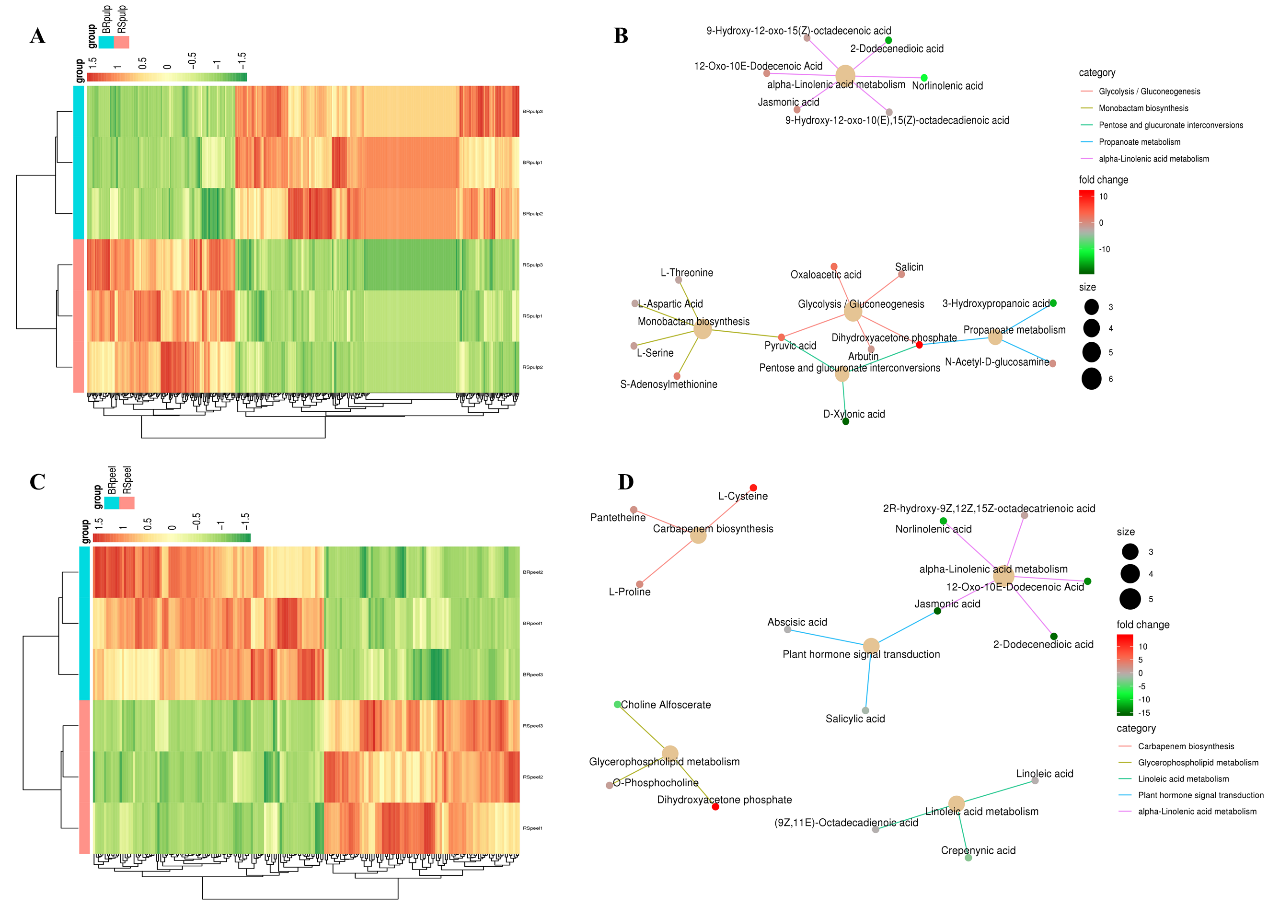


**Figure S8 Cluster heat maps and KEGG annotation of Mca fruit pulp and peel comparing the breaker stage (BR) vs ripening stage (RS) groups.** A cluster heat map of BR pulp vs RS pulp; B KEGG pathway enrichment net-plots of BR pulp vs RS pulp; C cluster heat map of BR peel vs RS peel; D KEGG pathway enrichment net-plots of BR peel vs RS peel.

**A**


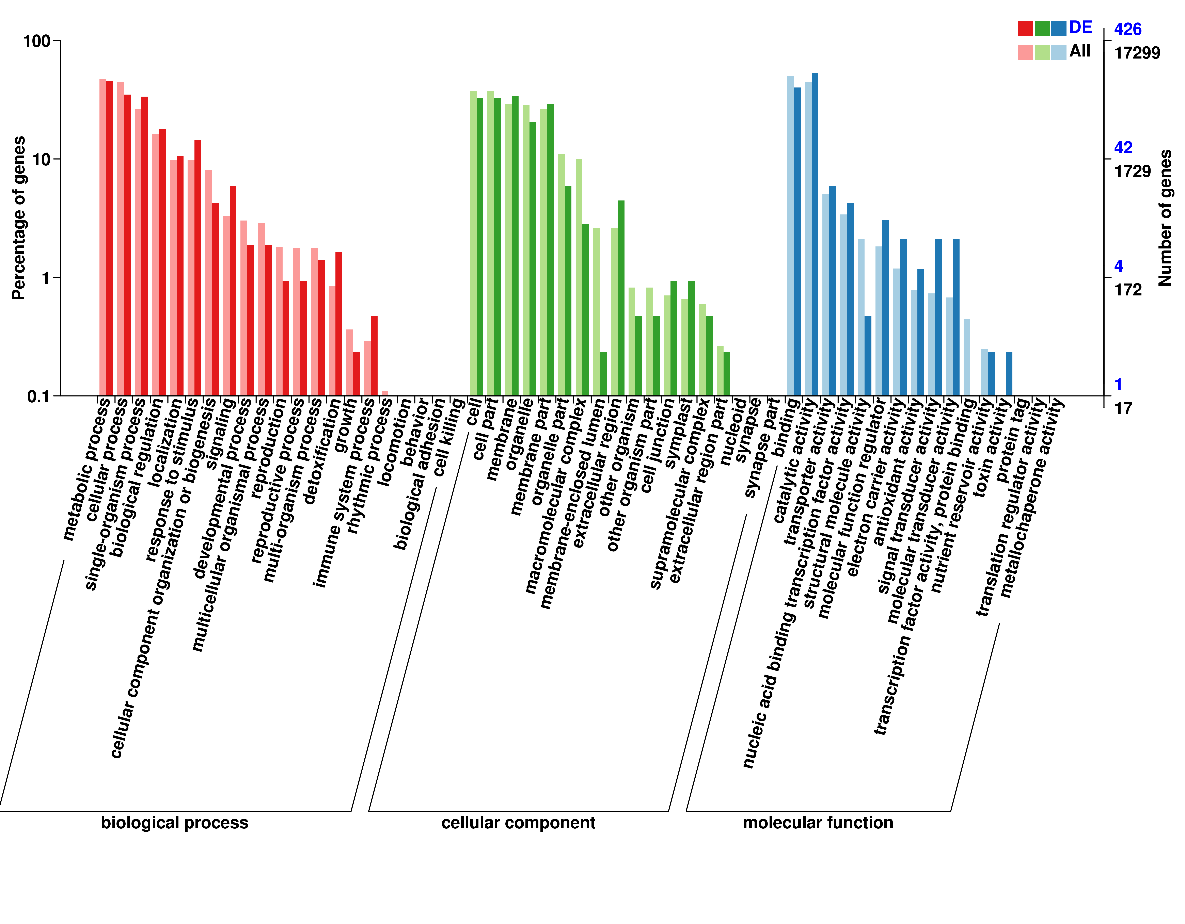


**B**


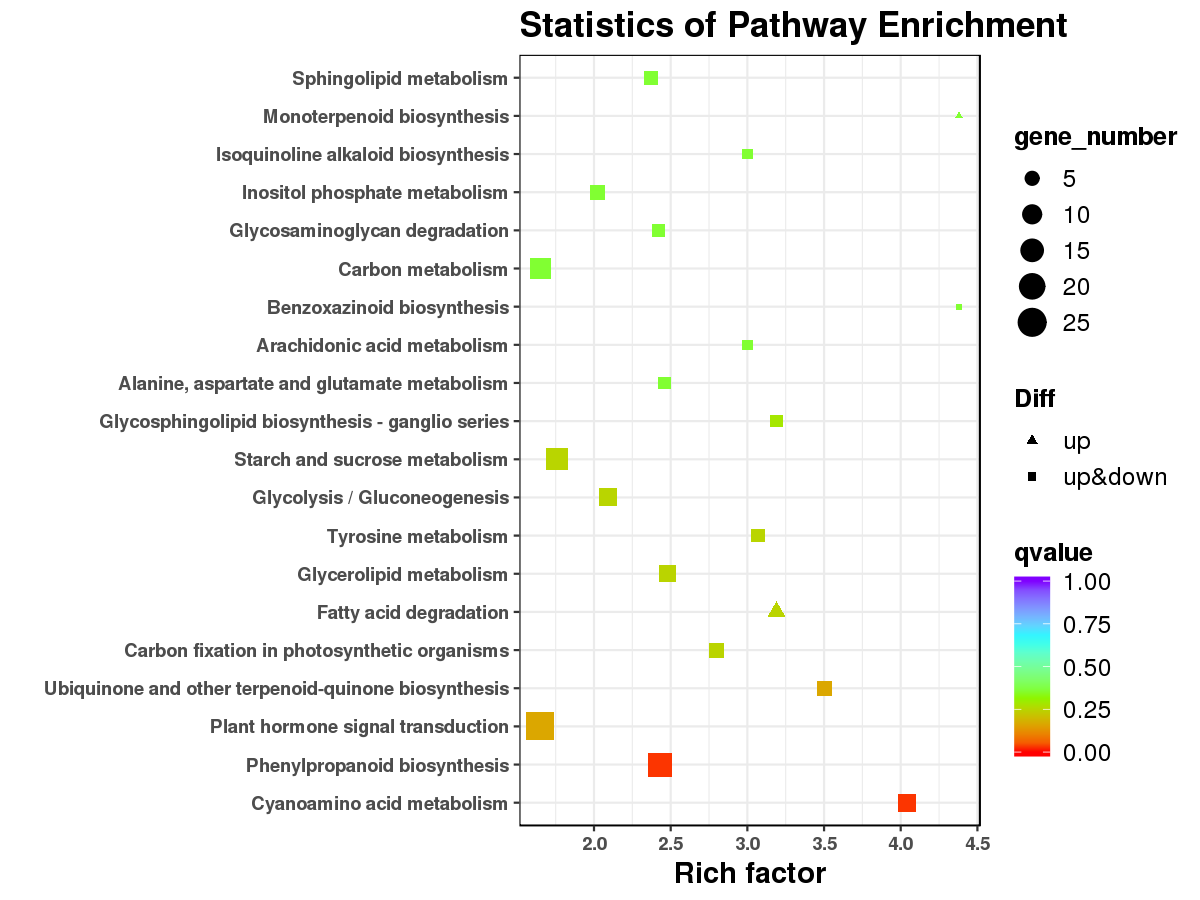


**Figure S9 GO enrichment (A) and KEGG annotation (B) analysis of peel samples in IM vs BR group.**

**A**


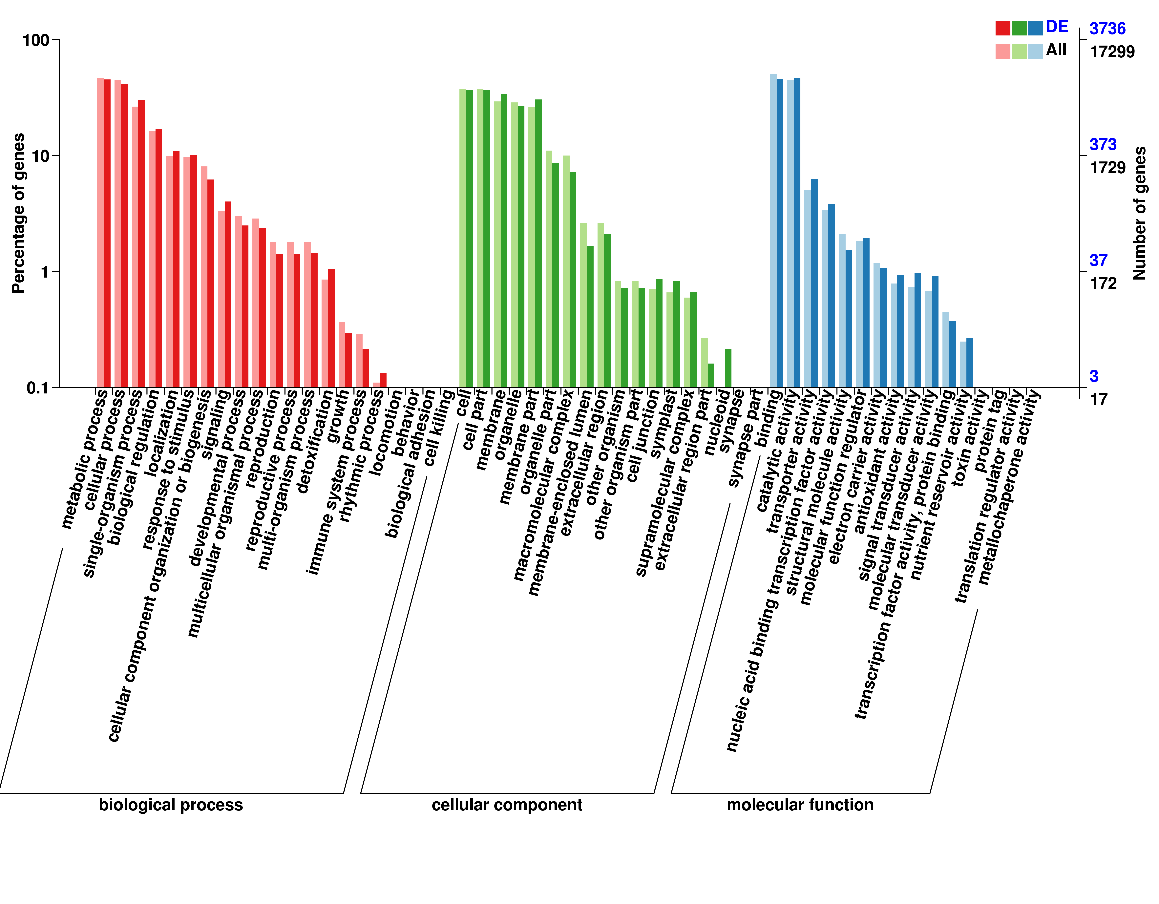


**B**


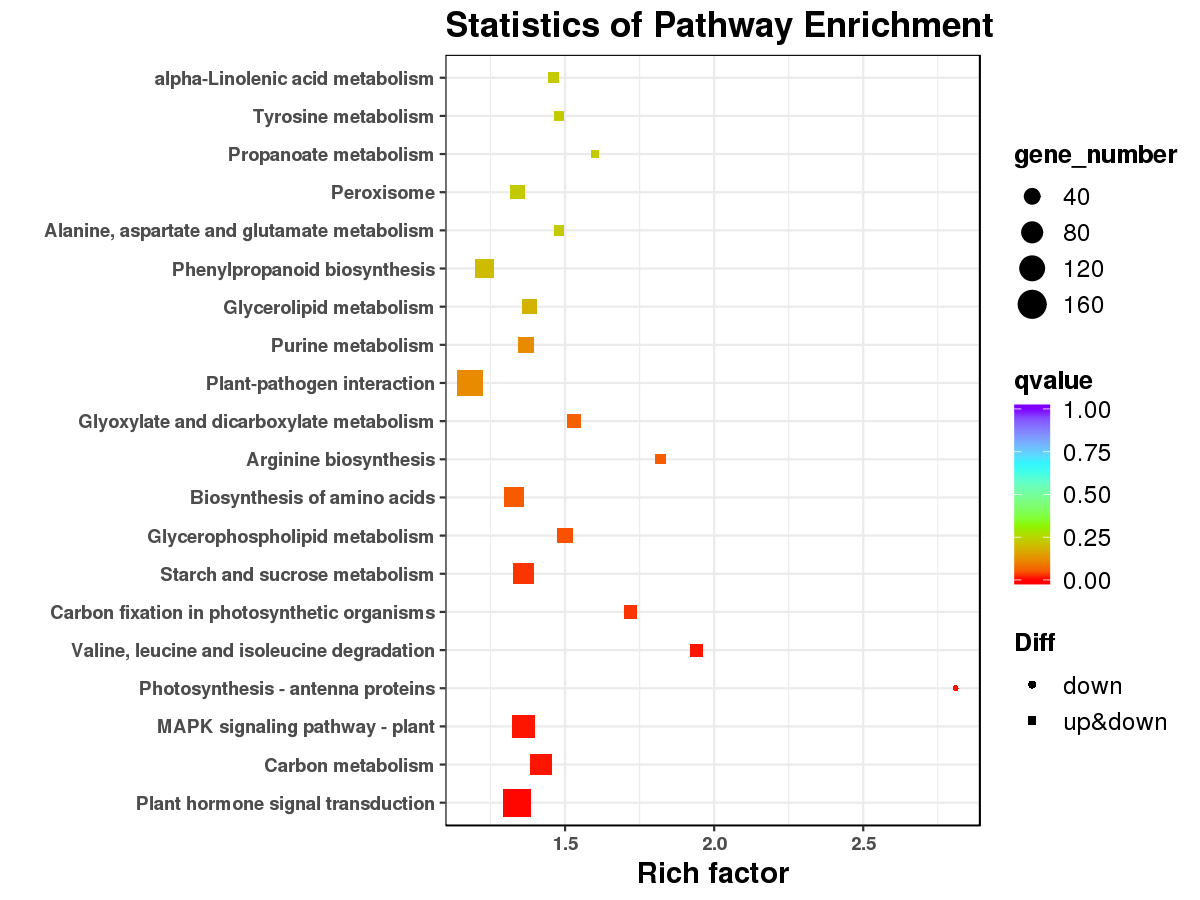


**Figure S10 GO enrichment (A) and KEGG annotation (B) analysis of peel samples in BR vs RS group.**

**A**


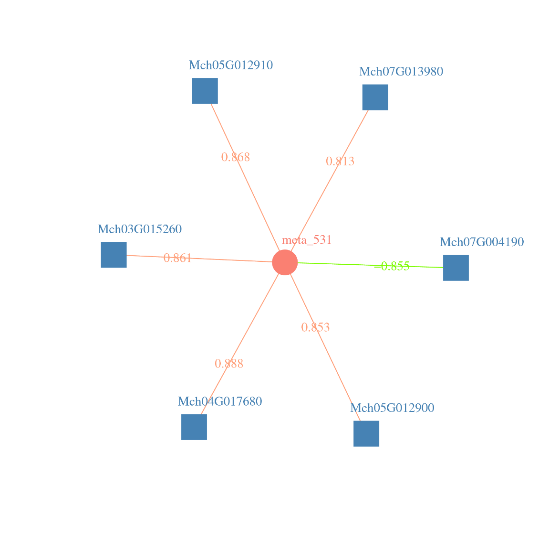


**B**


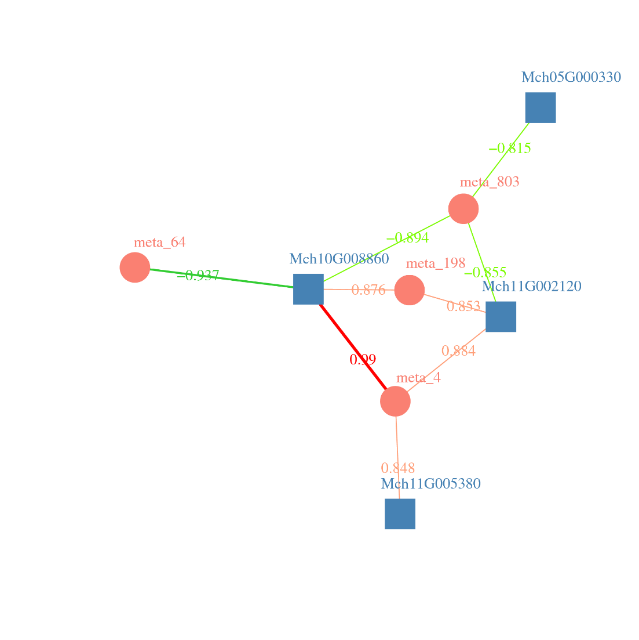


**C**

**
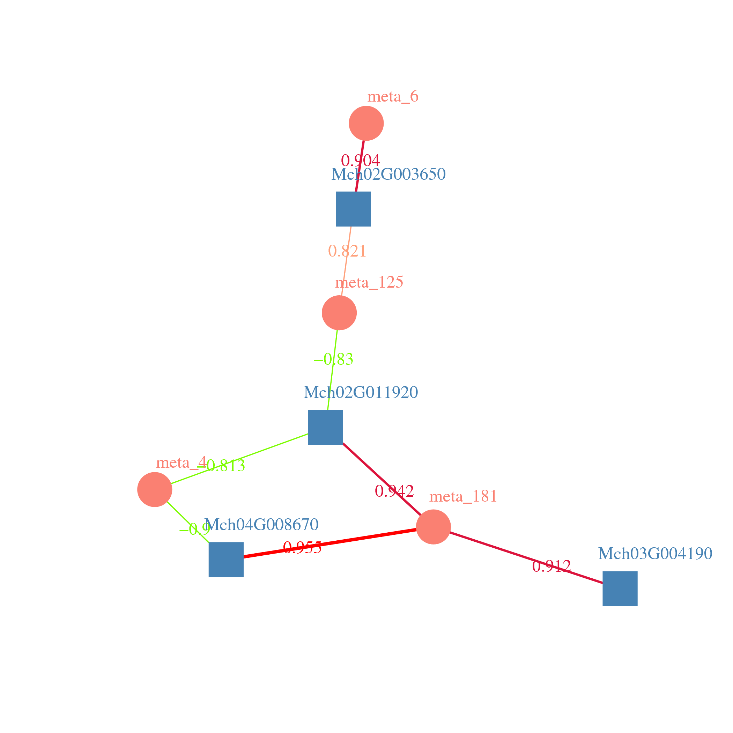
**

**Figure S11 A plant hormone signal transduction pathway (ko04075) network of DEMs and DEGs in IM vs BR group; B cysteine and methionine metabolism (ko00270) network of DEMs and DEGs in IM vs BR group; C glyoxylate and dicarboxylate metabolism (ko00630) network of DEMs and DEGs in IM vs BR group.**

**
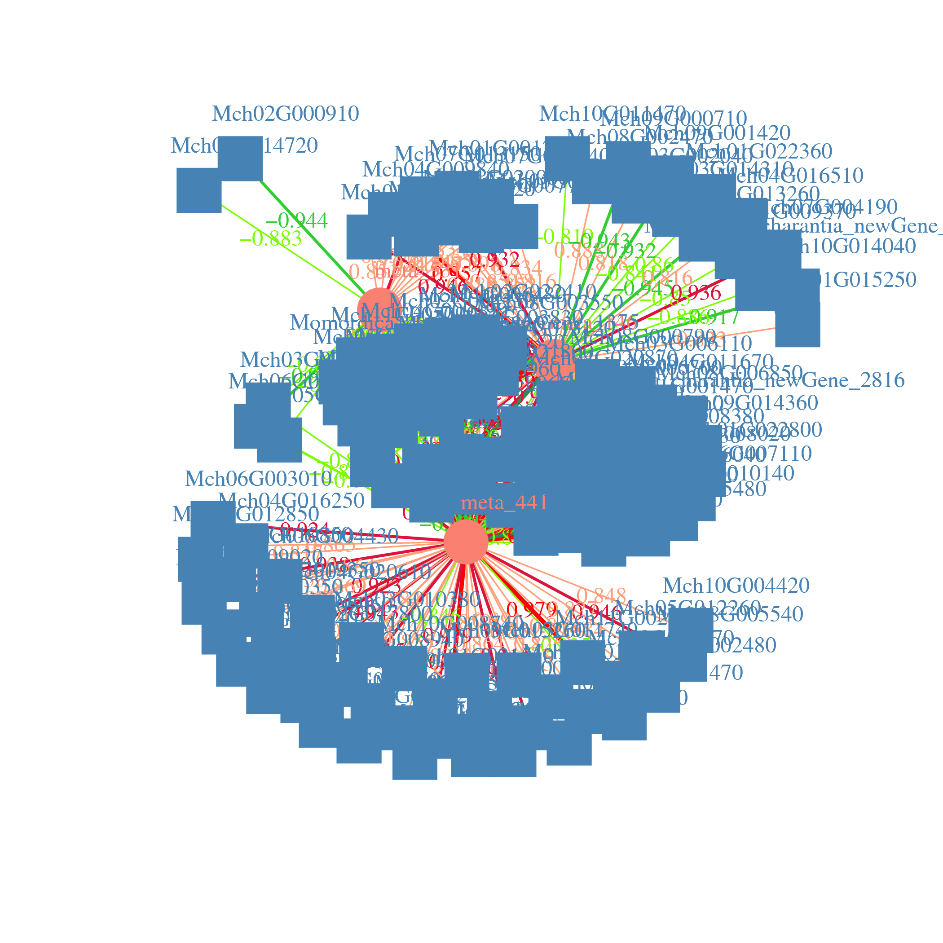
**

**B**

**
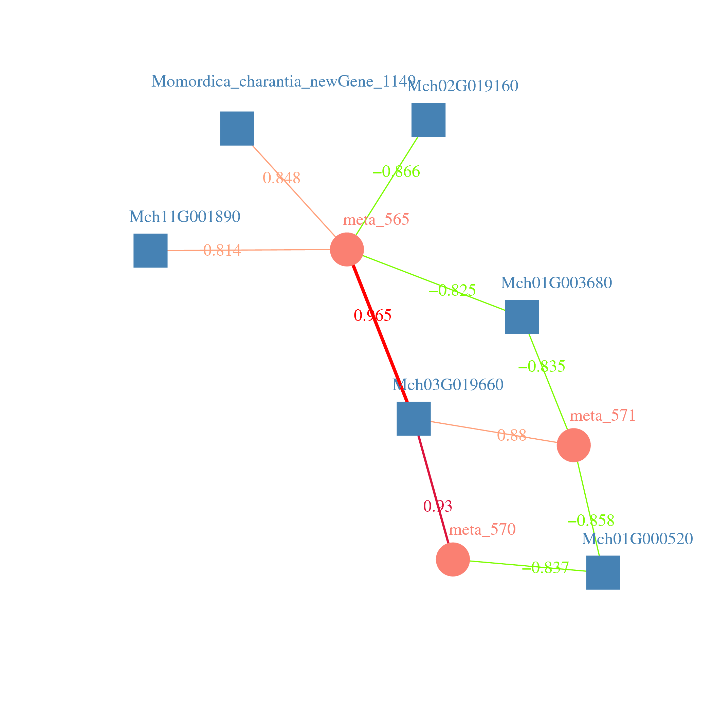
**

**C**


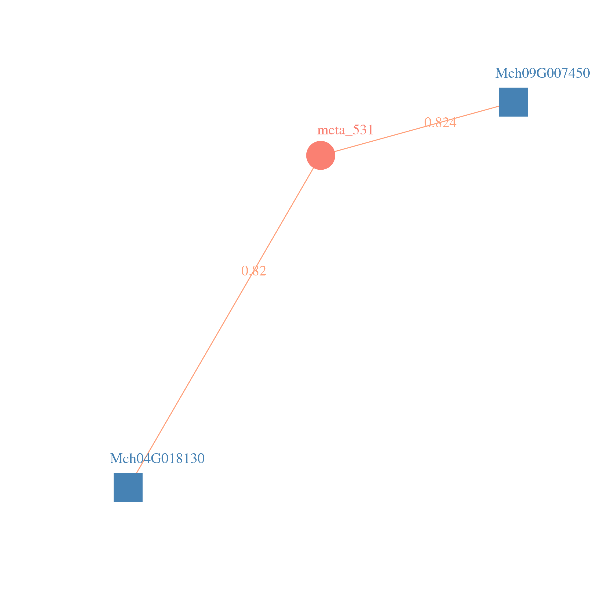


**Figure S12 A Plant hormone signal transduction pathway (ko04075) network of DEMs and DEGs in BR vs RS group; B linoleic acid metabolism pathway (ko00591) network of DEMs and DEGs in BR vs RS group; C carotenoid biosynthesis pathway (ko00906) network of DEMs and DEGs in BR vs RS group.**
